# The navigation for home service robot with the least power consumption

**DOI:** 10.1038/s41598-025-19760-7

**Published:** 2025-10-14

**Authors:** Yupei Yan

**Affiliations:** Department of Artificial Intelligence, Zhuhai City Polytechnic College, Zhuhai, 519090 China

**Keywords:** Home service robot, Least power consumption, Localization, Path planning, Navigation, Mechanical engineering, Electrical and electronic engineering

## Abstract

This paper discusses the navigation of the home service robot with least power consumption. Firstly, the localization of position of the robot with least power consumption is analyzed based on the geometrical relationship between robot and home appliance, then the localization of angle of the robot in the indoor environment with least power is proposed, which can be predicted by machine learning algorithms. Following, the path planning of the robot with least power is proposed, two supplements and optimizations of previous algorithms are proposed, the power consumption on the moving path is calculated according to the law of energy conservation, the conclusion is obtained that moving straight path and rotating in place will save more power than moving curve path. Then, the obstacle avoidance of the robot in the dynamic environment with least power consumption is proposed, the navigation process with least power consumption is analyzed and proposed in the indoor dynamic environment. Lastly, the experiment is conducted in the home to prove that compared with the DWA and TEB algorithm, there is least power consumption for the proposed navigation algorithm. The five novel points are summarized and proposed in the conclusion to save more power consumption in the localization of position and angle, path planning and navigation process of the robot.

**Supplementary Information:**

The online version contains supplementary material available at 10.1038/s41598-025-19760-7.

## Introduction

### Motivation

Home service robot is a special robot which can complete family service work, including movement, sensing, communication, controlling, executive, storage, interactive components, etc. Today, more and more home service robots are working in the house where is an indoor environment. The data from the Inertial Measurement Unit (IMU) sensors which are composed of the GPS, gyroscope, electronic compass, accelerometer is not accurate in the indoor environment. Some proposals are proposed to solve this issue, but no effective solution can be applied to solve it.

**Fig. 1:**
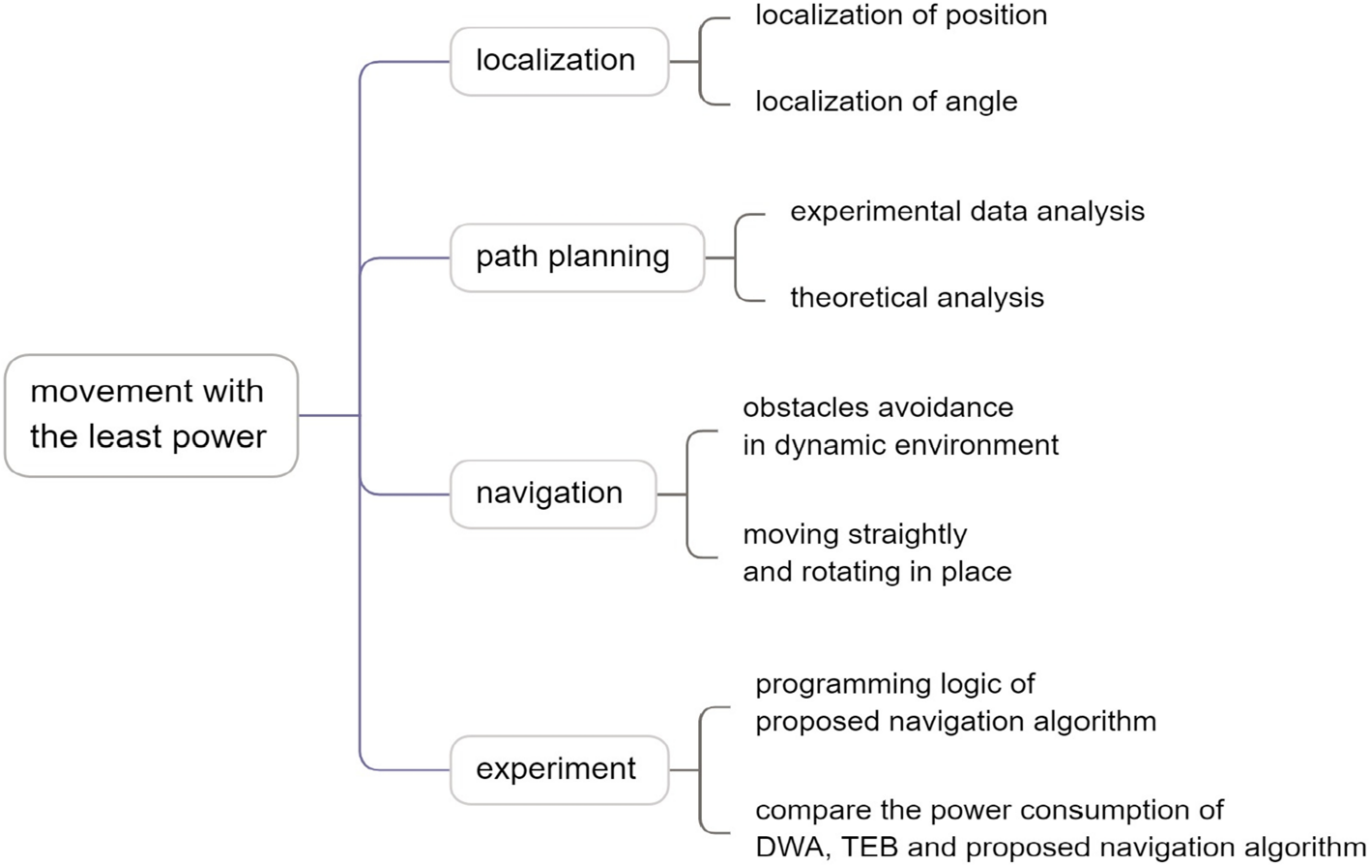
The structure of the article.

Another issue is how to save the power for the home service robot, because the home service robot needs to move for long time on the ground every day, which needs the supporting of a powerful battery. If the power consumption is large when the home service robot is working, then it will accelerate battery aging, and the home service robot needs to charge the power frequently, so the working efficiency of the home service robot becomes lower, which will bring trouble for the users and make users feel unsatisfied with the robot, and increasing the cost of the batteries, so how to save power is important for home service robot. The following schematic in Fig. 1 shows the brief structure of the article.

The movement of home service robot is driven by the wheels, usually the moving direction is forward, backward, right and left. The power will be saved a lot if the movement can be controlled more properly in the process of localization, path planning and navigation.

### Related work

In the existing research, lots of navigation algorithms of the mobile robots are proposed, which can be classified into two parts. One is classical navigation that includes the local navigation and global navigation. The other is navigation with artificial intelligence (AI). The following is the introduction to the related work of classical navigation.

One type of classical navigation is local navigation, the position and orientation of the mobile robot is determined by the equipped sensors, such as magnetic tape, inductive wire, magnetic spots, laser scanning sensor^1^. Usually the magnetic tape is placed on the ground which provides the magnetic field to guide the navigation of the mobile robot. Two dimension sensors array is constructed on the magnetic nail to be tracked by the mobile robot^2^, and the location and orientation were computed via sensor array data and hybrid computational optimization algorithms, but magnetic tape is limited to be applied in the static path and static environment^3^. The second method is the inductive wire method, which will generate magnetic field when current goes through the inductive wire, the navigation will be completed by judging the strength of magnetic field around the inductive wire under the ground, it can supply the accurate navigation schema, but the cost of installation of inductive wire under the ground and the subsequent maintenance will be high^4^. The third method is magnetic spot method, that the magnetic spot is embedded under the ground as a mark to guide the mobile robot to complete the navigation, the latest news about this method is that a deep-learning-based floor path model for navigation of mobile robot is proposed to improve the accuracy of the navigation^5^, but this method is suitable for the static environment and not applicable for the dynamic environment. The fourth method is laser guided navigation, deep reinforcement learning is proposed to realize the design of the system controller^6^, a dynamic matching algorithm based on the initial positioning value, which will reduce calculation time and the likelihood of mismatches occurring is proposed, the Taylor series iteration was also proposed by them in order to improve the positioning of the mobile robot^7^. Other than that, a robust stereo SLAM algorithm is proposed based on dynamic region rejection to reduce absolute trajectory error, because it is discovered that the localization and mapping accuracy using visual SLAM (vSLAM) in a dynamic environment is significantly lower than in a static environment, due to inaccurate data association caused by dynamic or moving objects, this novel algorithm is proposed^8^. Additionally, it has been demonstrated that using this SLAM navigation methodology in conjunction with bio-inspired methodologies will result in an improved navigation method. Then, the fifth method is proposed, Dai conducted research using SLAM-based for robot navigation and localization algorithms by proposing an attitude estimation algorithm based on Kalman filter (KF) information fusion from vision SLAM^9^.

For global navigation, the heuristics graph search algorithm is proposed which is paths finding methods. An A-star (A*), D-star Lite (D* Lite) and Dijkstra are some of these methods. The first is A* algorithm, lots of improved A* algorithms are proposed, a time window is proposed based on A* to solve the conflict-free path planning problem for the navigation of mobile robot. The second method is D* Lite algorithm, D* Lite algorithm can plan the shorter path and plan the path faster in large areas, whereas the A* algorithm may be more effective than the D* Lite algorithm in static and small area work environments^10^. A novel planning architecture to overcome the shortcomings of existing kinematic and artificial potential field-based path planning methods are proposed, the cubic Bezier curve is adopted in order to smooth the robot’s path^11^. Latest, the D* Lite algorithm in path planning in autonomous exploration based on multi-criteria decision-making (MCDC) strategies is proposed to improve the accuracy of the navigation^12^. The third method is Dijkstra which can find the shortest path between two top points in the graph, a modified Dijkstra algorithm is proposed that several different environments are simulated as part of a parameter algorithm in order to find the best time and velocity for robot movement^13^. The Dijkstra algorithm can be combined with the Dynamic Window Approach (DWA) in path planning and obstacle avoidance in the navigation of the mobile robot. For global navigation, there are many heuristic search algorithms for finding the shortest path are proposed if given a graph, in which a graph search algorithm approach is more commonly used in environments with no predefined paths and where a robot can freely move in the free configuration space. When the workspace changes and obstacles appear unexpectedly, only local and global navigation must be retrained to carry out data collection activities and follow the new or updated pseudo code. It needs to be combined with other techniques to ensure intelligent and optimal operation, especially in a dynamic environment^14^.

Artificial intelligence (AI) algorithm is developed to solve problems in the navigation of mobile robot^15^, the first method is Fuzzy Logic (FL) algorithm, they are capable of recognizing, representing, manipulating, deciphering, and exploiting ambiguous and uncertain facts and information. The FL controller approach to teach the mobile robot to track and follow a path under various loads^16^, also the Mamdani method is simulated which is available in FL to navigate an AGV based on speed and distance towards the obstacle in order to achieve smooth and precise stopping during operation. The second method is neural network (NN), also known as an artificial neural network (ANN) or simulated neural network (SNN)^17^, in order to overcome the problems of slow convergence speed, the highest number of iterations, and unstable convergence performance in the Q-learning algorithm by improving the NN algorithm for path planning, the improved NN algorithm increases the convergence speed with better effect compared to the traditional Q-learning algorithm^18^. While a hybrid intelligent real-time optimal control approach is proposed based on deep neural networks (DNN) in order to improve the autonomy and intelligence of AGVs in navigation control^19^. The third method is The Particle Swarm Optimization (PSO), a PSO optimization is proposed to avoid path conflict and crashes^20^, another improved PSO (IPSO) was also introduced by the same research team in order to improve the slow convergence speed, poor optimization result, and incomplete search during mobile robot path planning by using the classical navigation method. The fourth method is The Genetic Algorithm (GA), an improved adaptive GA combining with simulated annealing is proposed in their work for path planning to achieve a strong ability to avoid local optima and faster convergence speed^21^, another improved GA is proposed to achieve efficient path planning capabilities in complicated maps for mobile robots which use of the A* algorithm evaluation function, also this GA is applied with other algorithms in their studies to be applied in path planning, like Multi-Population Migration Genetic Algorithm (MPMGA) , and GA are used to obtain the control points of the Bezier curve to solve the problem of redundant nodes and peak inflection points in the path planning process of traditional algorithms^22^.

### Proposed method

The above methods have achieved lots of improvements of the navigation of the mobile robot, which aims to the shortest path or shortest time of navigation. However, there is relatively little research on the least power consumption of the navigation. Therefore, this paper discusses how to save the power in the process of location, path planning and navigation of the home service robot in the indoor and dynamic environment, in order to complete the navigation with least power consumption, the experiment is conducted and the experimental data is analyzed, the innovation of this paper can be listed as following:

The geometrical position between robot and home appliance is calculated to obtain the location of position of the robot, no need to calculate large quantity of values of data frequently in each second in SLAM algorithm, thus saving lots of power consumption of CPU and RAM in the robot.

The localization of angle of home service robot is predicted by three machine learning algorithms in the indoor environment, also avoiding the calculation of the large quantity data of SLAM algorithm and saving the power consumption of CPU and RAM.

The next innovation is that from theoretical analysis and experimental data processing, it can be concluded that moving on straight path and rotating in place will save more power than moving on curve path.

The following innovation is that compared with previous algorithms, two innovations of data processing are proposed, the first is to obtain the power consumption that only for the movement of the robot by judging the power consumption when robot is in stopped status, the second is that the noise data is judged and removed before selecting the abnormal data in the database.

The fifth innovation is that the navigation algorithm with least power consumption is proposed, in which the obstacles avoidance in the dynamic environment, the computation of the angle between robot and destination, the iterated navigation process is analyzed based on the result from the above innovation of localization and path planning algorithm.

The architecture of this paper is as follows. Firstly, Section "Localization with Least power" introduces the location of position and location of angle for the robot with least power. Secondly, Section "Path planning with the least power consumption." compared the power consumption of different paths from the theoretical and experimental analysis, and a conclusion is obtained that moving along straight path and rotating in place will save more power than moving along curve path. Then, the navigation with least power is proposed in the indoor and dynamic environment in Section "Navigation with least power consumption.". The experiment is conducted to compare the power consumption of DWA, TEB and the proposed navigation algorithm to finish the same navigation task in the home, the result shows that the proposed navigation algorithm will save more power in Sect. 5, Finally, Section "Conclusion." is the conclusion of this paper.

## Localization with least power

The localization contains two parts, one is the localization of position, that robot should know where itself located in the environment, the other is the localization of angle, that the robot should know which direction it points to, the following will discuss the issue that how to obtain the localization of position with least power.

### The localization of position of home service robot with least power consumption

The general algorithms of localization of position are proposed based on the computation of large number of feature data extraction and matching algorithms from the built maps, which are computed frequently every second and consume lots of CPU and RAM resources, and this will leads to high power consumption, in order to reduce the power consumption in the process of localization of position, the other algorithms of localization of position should be proposed.

#### The localization of position based on the location of home appliances

**Fig. 2:**
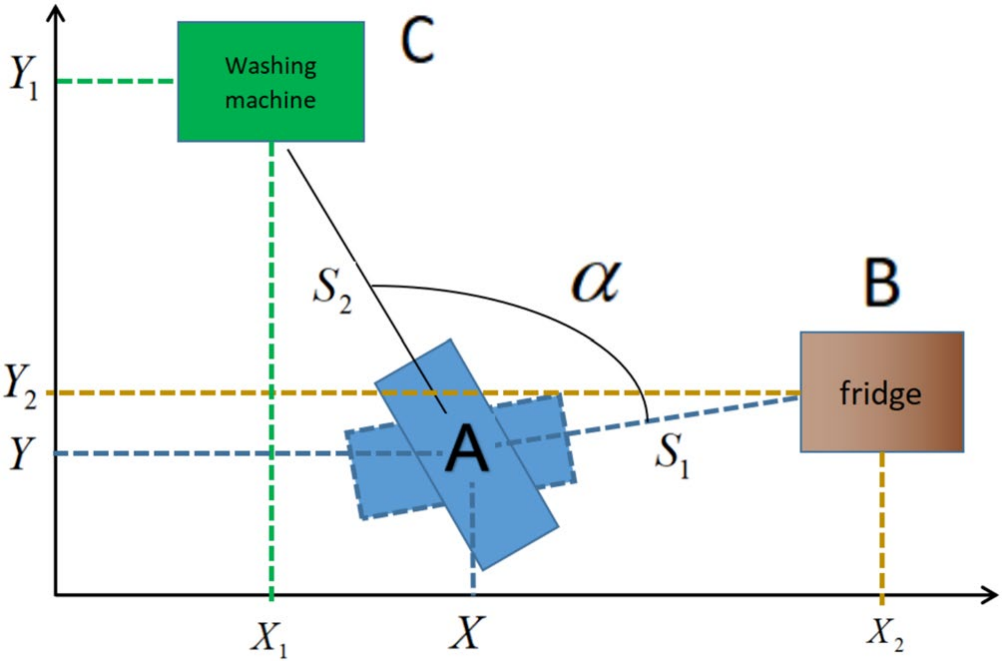
The relative positions of home service robot and home appliances.

In Fig. 2, the home service robot is located at position A after the movement for a while, the fridge is located at position B, and the washing machine is located at position C. The coordinate values of B and C can be obtained from the map.

**Fig. 3:**
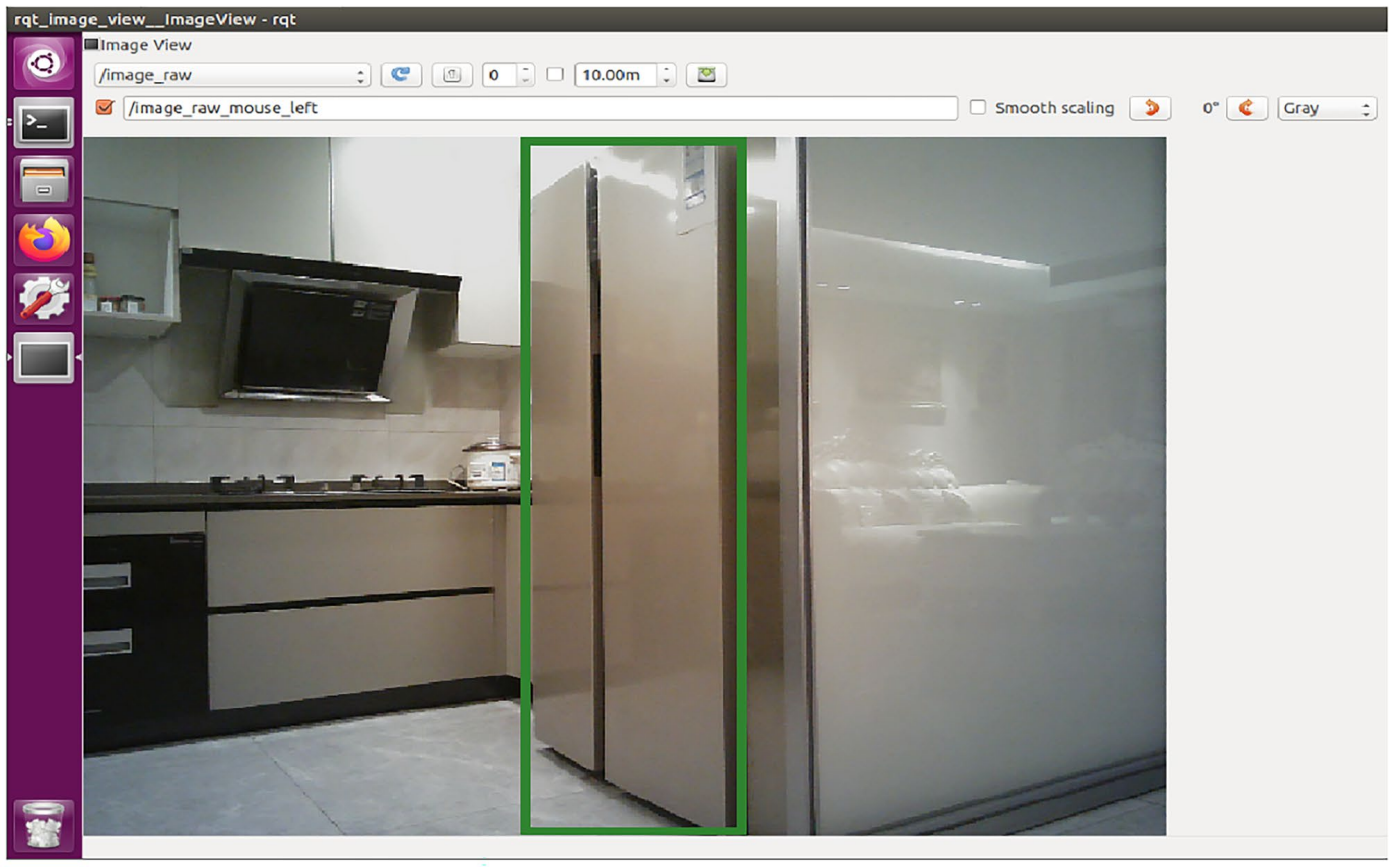
The robot rotates until the fridge is in the center of the video in ROS.

There is camera on the robot and the video can be outputted and displayed from the camera topic in the ROS (Robot Operating System). The robot rotates until the fridge is in the center of the video, which can be realized from the following calculation: the green rectangle can be drawn in the frames of the outputted video with Open CV library as Fig. 3 shows, if the coordinate value of the top left point of the green rectangle is ( x1, y1), the coordinate value of the bottom right point of the green rectangle is (x 2, y2), if the resolution of the video is 600*400, and half the length of the horizontal axis is 600/2 = 300, if the allowed error is 1%, then when (x 1 +  x 2) /2 < 303 while (x 1 +  x 2) /2 > 297, the fridge is in the center of the video, which indicates that the robot is facing positively the fridge at this moment, otherwise, the robot should continue to rotate to adjust the angle until the condition is satisfied.

**Fig. 4:**
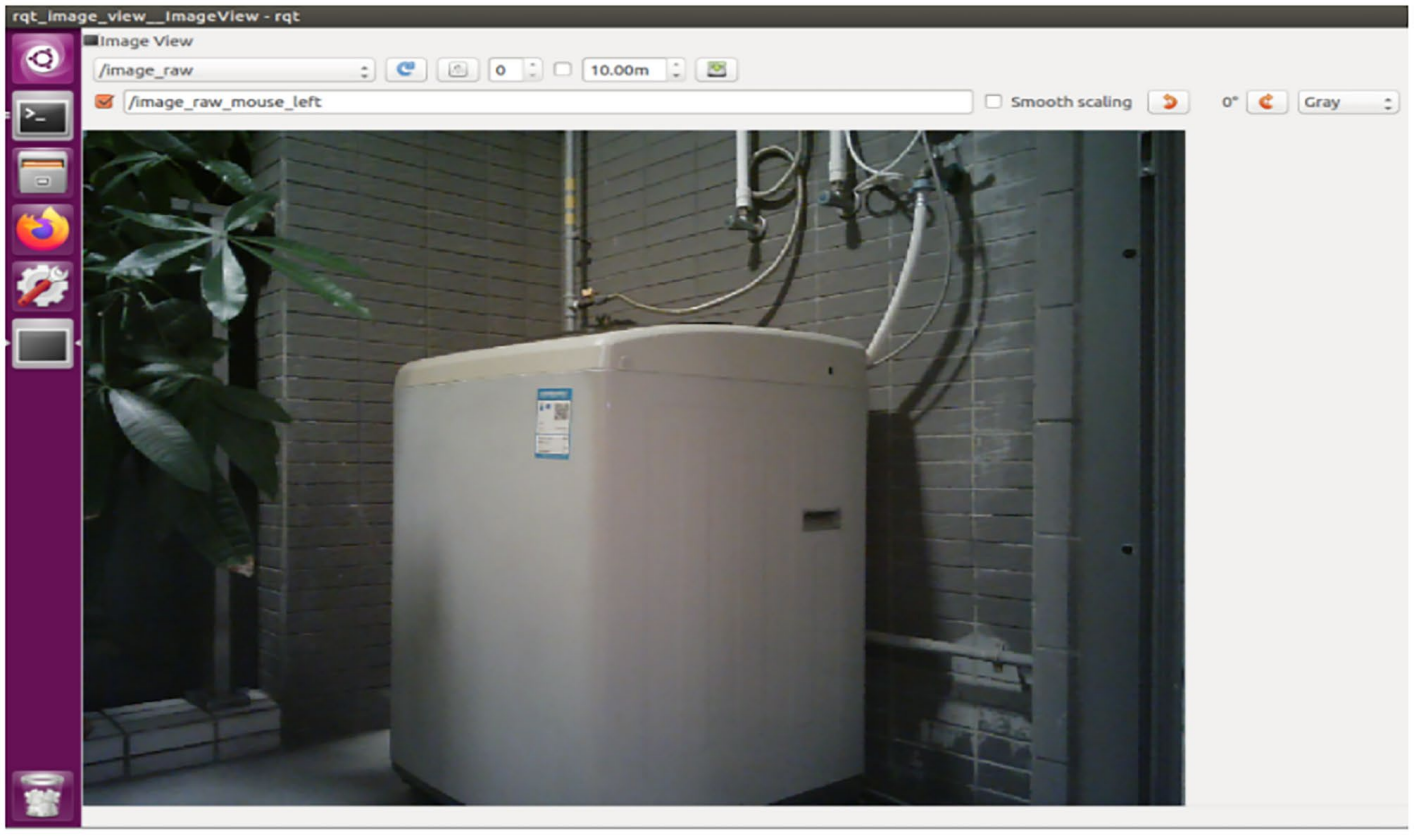
The robot rotates until the washing machine is in the center of the video.

When the fridge is in the center of the video, the robot will record the angle value at this moment as β, then it will stay in this position and rotate until it finds another home appliance, such as the washing machine in Fig. 4, where the robot’s angle value can be recorded as γ, so the angle α in Fig. 2 is α = γ-β. α can be obtained from the outputted data of the /odom topic in ROS or from the outputted data of laser scan sensor.

#### The angle of rotation of home service robot

**Fig. 5:**
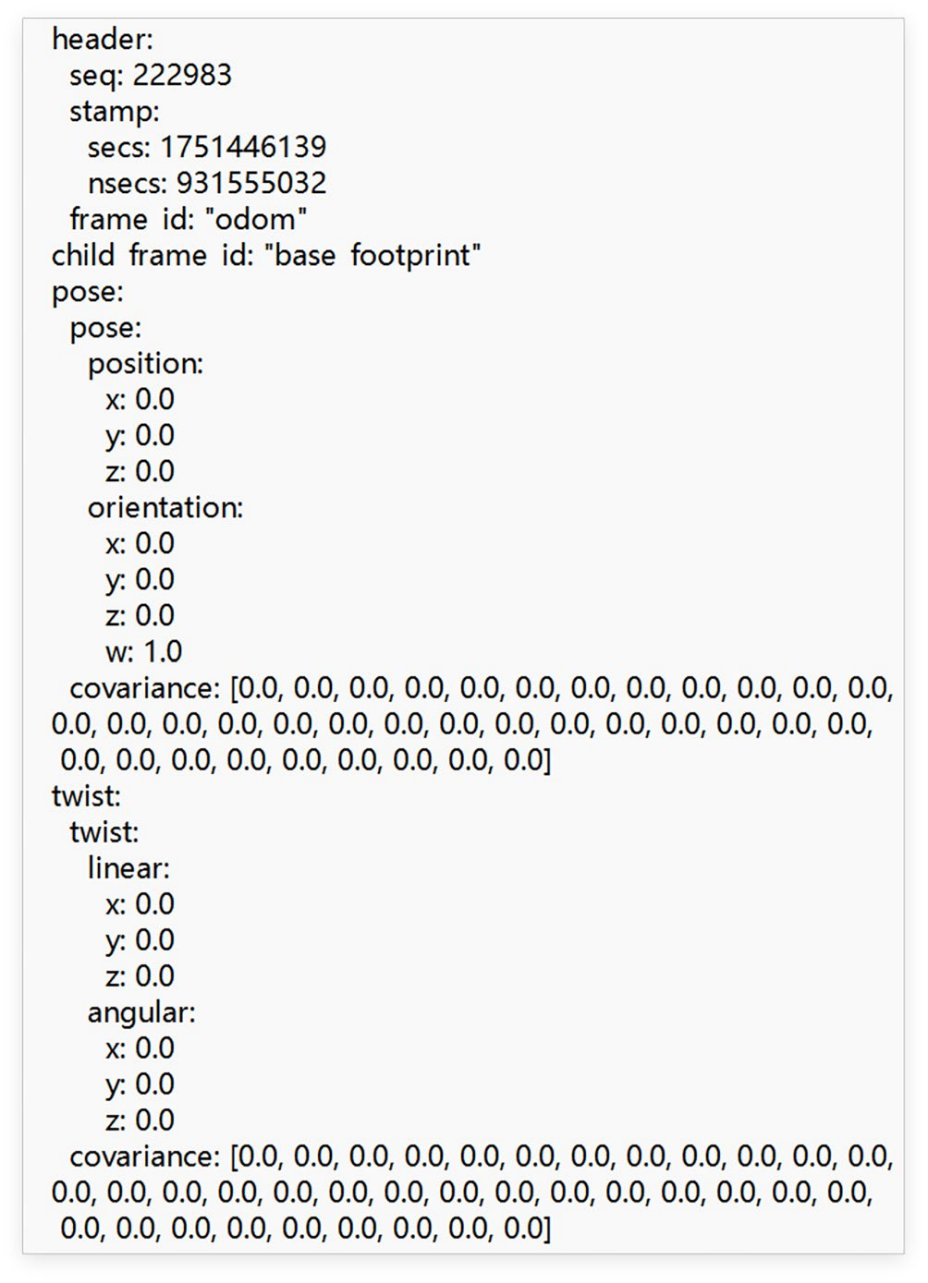
Odometer data outputted from /odom topic in ROS.

In ROS, odometer data can be outputted from the /odom topic as Fig. 5 shows, which can be recorded to the file “odom.txt” by inputting command "rostopic echo /odom > odom. txt" in Ubuntu . In Fig. 5, if the robot moves on the x–y surface, the value of z in the orientation field is the angle of rotation of the robot.

In Fig. 5, the value of each data field can be extracted and be exported to the tables in database as Table 1 shows. The relationship between the key in Table 1 and the data field in the Fig. 5 is following, the key second, px, py, pz, ox, oy, oz, ow, lx, ly, lz, ax, ay, az in Table 1 corresponds to the data filed secs, position x, position y, position z, orientation x, orientation y, orientation z, orientation w, linear x, linear y, linear z, angular x, angular y, angular z in Fig. 5.

**Table 1:** The data table of odometer data from /odom topic.

Second	px	py	pz	ox	oy	oz
1,658,817,762	− 1.916613633	− 0.875847304	0	0	0	− 0.147809411
1,658,817,762	− 1.916613633	− 0.875847304	0	0	0	− 0.147809411
1,658,817,762	− 1.916613633	− 0.875847304	0	0	0	− 0.147809411
1,658,817,762	− 1.916613633	− 0.875847304	0	0	0	− 0.147809411
1,658,817,762	− 1.916613633	− 0.875847304	0	0	0	− 0.147809411
1,658,817,762	− 1.916613633	− 0.875847304	0	0	0	− 0.147809411
1,658,817,762	− 1.916613633	− 0.875847304	0	0	0	− 0.147809411
1,658,817,762	− 1.916613633	− 0.875847304	0	0	0	− 0.147809411
1,658,817,762	− 1.916613633	− 0.875847304	0	0	0	− 0.147809411
1,658,817,762	− 1.916613633	− 0.875847304	0	0	0	− 0.147809411
1,658,817,762	− 1.916613633	− 0.875847304	0	0	0	− 0.147809411
1,658,817,762	− 1.916613633	− 0.875847304	0	0	0	− 0.147809411
1,658,817,762	− 1.916613633	− 0.875847304	0	0	0	− 0.147809411
1,658,817,762	− 1.916613633	− 0.875847304	0	0	0	− 0.147809411
1,658,817,762	− 1.916613633	− 0.875847304	0	0	0	− 0.147809411
1,658,817,762	− 1.916613633	− 0.875847304	0	0	0	− 0.147809411
1,658,817,762	− 1.916613633	− 0.875847304	0	0	0	− 0.147809411
1,658,817,762	− 1.916613633	− 0.875847304	0	0	0	− 0.147809411

Second	ow	lx	ly	lz	ax	ay	az
1,658,817,762	0.989015863	0	0	0	0	0	0
1,658,817,762	0.989015863	0	0	0	0	0	0
1,658,817,762	0.989015863	0	0	0	0	0	0
1,658,817,762	0.989015863	0	0	0	0	0	0
1,658,817,762	0.989015863	0	0	0	0	0	0
1,658,817,762	0.989015863	0	0	0	0	0	0
1,658,817,762	0.989015863	0	0	0	0	0	0
1,658,817,762	0.989015863	0	0	0	0	0	0
1,658,817,762	0.989015863	0	0	0	0	0	0
1,658,817,762	0.989015863	0	0	0	0	0	0
1,658,817,762	0.989015863	0	0	0	0	0	0
1,658,817,762	0.989015863	0	0	0	0	0	0
1,658,817,762	0.989015863	0	0	0	0	0	0
1,658,817,762	0.989015863	0	0	0	0	0	0
1,658,817,762	0.989015863	0	0	0	0	0	0
1,658,817,762	0.989015863	0	0	0	0	0	0
1,658,817,762	0.989015863	0	0	0	0	0	0
1,658,817,762	0.989015863	0	0	0	0	0	0

The statistical values of data in Table 1 is shown in Table 2, it shows that the changes of position in horizontal direction of the robot seems more obviously than it in vertical direction from the values of std_deviation in Table 2. On the contrary, the velocity of robot in vertical direction changes more obviously than it in horizontal direction. It is the 95% confidence intervals in Table 2, from the values in row “margin_of_error/mean” in Table 2, it is obvious that the changes of values in velocity is more dramatically than changes of values in position.

**Table 2:** The statistical values of data in Table 1.

	Second	px	py	pz	ox	oy	oz	ow
Mean	1,691,290,796.3438792	− 1.534765244	− 1.047930972	0	0	0	0.103708335213949	0.6287238812074727
Variance	293,286.95161726035	3.6170685516797274	1.5184193359749385	0	0	0	0.4957462696363507	0.09820459276980015
Std_deviation	541.5597396569102	1.9018592355060686	1.2322415899388148	0	0	0	0.7040925149696954	0.31337612029285217
Confidence_interval_0.025	1,691,290,792.6138053	− 1.547864585	− 1.05641822	0	0	0	0.0988587925243403	0.6265654562264465
Confidence_interval_0.975	1,691,290,800.0739532	− 1.521665904	− 1.039443723	0	0	0	0.1085578779035577	0.6308823061884988
Margin_of_error/mean	2.2054597918636533e− 09	− 0.008535078	− 0.008099053				0.04676135895542202	0.0034330252842963336

	Second	lx	ly	lz	ax	ay	az
Mean	1,691,290,796.3438792	− 0.008015634	0.0001951370108299683	0	0	0	− 0.016431692
Variance	293,286.95161726035	0.13556351474103517	1.6612361784604685e− 05	0	0	0	0.09578679355017886
Std_deviation	541.5597396569102	0.36818950927618127	0.004075826515518624	0	0	0	0.3094944160242295
Confidence_interval_0.025	1,691,290,792.6138053	− 0.010551594	0.00016706414503150517	0	0	0	− 0.018563381
Confidence_interval_0.975	1,691,290,800.0739532	− 0.005479673	0.00022320987662843145	0	0	0	− 0.014300003
Margin_of_error/mean	2.2054597918636533e− 09	− 0.316376784	0.14386233384974972				− 0.129730349

There are about 50 rows in one second in Table 1, so the data in Table 1 can be averaged in one second and take the key “second” as the primary key in database as Table 3 shows.

**Table 3:** The odometer data in each second.

Second	px	py	pz	ox	oy	oz	ow	lx	ly	lz	ax	ay	az
1,691,289,812	− 0.064948742	− 0.409979159	0	0	0	− 0.809016994	0.587785252	0	0	0	0	0	0
1,691,289,813	− 0.064948742	− 0.409979159	0	0	0	− 0.809016994	0.587785252	0	0	0	0	0	0
1,691,289,814	− 0.064948742	− 0.409979159	0	0	0	− 0.809016994	0.587785252	0	0	0	0	0	0
1,691,289,815	− 0.064948742	− 0.409979159	0	0	0	− 0.809016994	0.587785252	0	0	0	0	0	0
1,691,289,816	− 0.064948742	− 0.409979159	0	0	0	− 0.809016994	0.587785252	0	0	0	0	0	0
1,691,289,817	− 0.064949722	− 0.409982176	0	0	0	− 0.809016994	0.587785252	0.00016	0	0	0	0	8e− 05
1,691,289,818	− 0.115638127	− 0.564948065	0	0	0	− 0.810750493	0.58537727145446	0.28578	8e− 05	0	0	0	− 0.00088
1,691,289,819	− 0.158213113	− 0.68890615	0	0	0	− 0.808810589	0.5880667536703599	0.021879999999999997	0.0008	0	0	0	0.010959999999999994
1,691,289,820	− 0.143509438	− 0.627549311	0	0	0	− 0.786686837	0.61706287098312	− 0.16102	− 0.00088	0	0	0	0.12041999999999999
1,691,289,821	− 0.114134145	− 0.455068063	0	0	0	− 0.744415851	0.6674572394846401	− 0.19116	− 0.00064	0	0	0	0.11963999999999998
1,691,289,822	− 0.106861958	− 0.216829638	0	0	0	− 0.697028122	0.7167697696054001	− 0.27274	0.00024	0	0	0	0.12289999999999997
1,691,289,823	− 0.126755151	0.02187789802805419	0	0	0	− 0.651639977	0.75840469445016	− 0.14368	0.00016	0	0	0	0.06581999999999998
1,691,289,824	− 0.131185039	0.052635177314834004	0	0	0	− 0.645835545	0.76343224764978	0.07314	0.0010400000000000001	0	0	0	− 0.05536
1,691,289,825	− 0.101356816	− 0.234587799	0	0	0	− 0.691455116	0.72204993982858	0.29552	− 0.00056	0	0	0	− 0.12578
1,691,289,826	− 0.105215073	− 0.470436822	0	0	0	− 0.724807927	0.6887789033661601	0.45549999999999996	0.0030399999999999997	0	0	0	− 0.08218

Second	px	py	pz	ox	oy	oz	ow	lx	ly	lz	ax	ay	az
1,691,289,812	− 0.064948742	− 0.409979159	0	0	0	− 0.809016994	0.587785252	0	0	0	0	0	0
1,691,289,813	− 0.064948742	− 0.409979159	0	0	0	− 0.809016994	0.587785252	0	0	0	0	0	0
1,691,289,814	− 0.064948742	− 0.409979159	0	0	0	− 0.809016994	0.587785252	0	0	0	0	0	0
1,691,289,815	− 0.064948742	− 0.409979159	0	0	0	− 0.809016994	0.587785252	0	0	0	0	0	0
1,691,289,816	− 0.064948742	− 0.409979159	0	0	0	− 0.809016994	0.587785252	0	0	0	0	0	0
1,691,289,817	− 0.064949722	− 0.409982176	0	0	0	− 0.809016994	0.587785252	0.00016	0	0	0	0	8e− 05
1,691,289,818	− 0.115638127	− 0.564948065	0	0	0	− 0.810750493	0.58537727145446	0.28578	8e− 05	0	0	0	− 0.00088
1,691,289,819	− 0.158213113	− 0.68890615	0	0	0	− 0.808810589	0.5880667536703599	0.021879999999999997	0.0008	0	0	0	0.010959999999999994
1,691,289,820	− 0.143509438	− 0.627549311	0	0	0	− 0.786686837	0.61706287098312	− 0.16102	− 0.00088	0	0	0	0.12041999999999999
1,691,289,821	− 0.114134145	− 0.455068063	0	0	0	− 0.744415851	0.6674572394846401	− 0.19116	− 0.00064	0	0	0	0.11963999999999998
1,691,289,822	− 0.106861958	− 0.216829638	0	0	0	− 0.697028122	0.7167697696054001	− 0.27274	0.00024	0	0	0	0.12289999999999997
1,691,289,823	− 0.126755151	0.02187789802805419	0	0	0	− 0.651639977	0.75840469445016	− 0.14368	0.00016	0	0	0	0.06581999999999998
1,691,289,824	− 0.131185039	0.052635177314834004	0	0	0	− 0.645835545	0.76343224764978	0.07314	0.0010400000000000001	0	0	0	− 0.05536
1,691,289,825	− 0.101356816	− 0.234587799	0	0	0	− 0.691455116	0.72204993982858	0.29552	− 0.00056	0	0	0	− 0.12578
1,691,289,826	− 0.105215073	− 0.470436822	0	0	0	− 0.724807927	0.6887789033661601	0.45549999999999996	0.0030399999999999997	0	0	0	− 0.08218

**Fig. 6:**
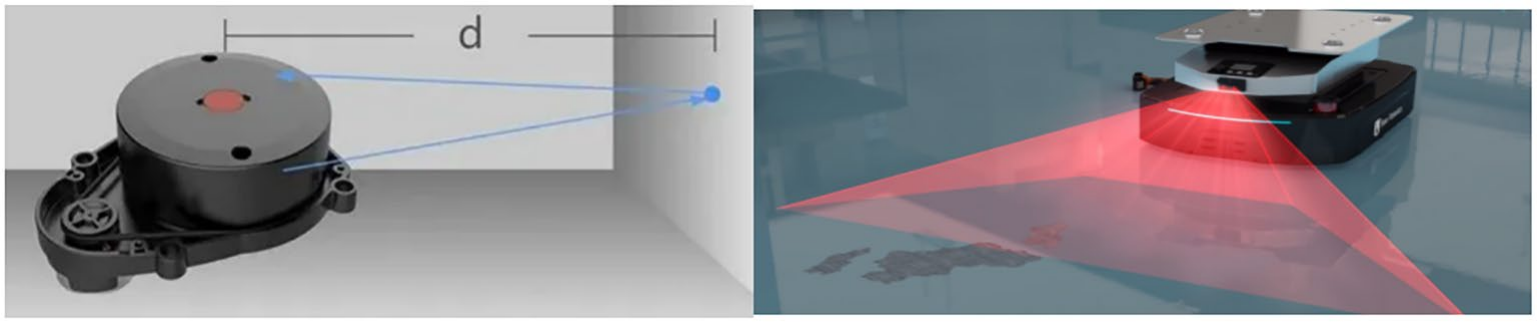
The distance of the obstacles can be measured by laser scan sensor.

The angle of rotation α can be obtained from the outputted data of /odom topic in ROS, it also can be obtained from the laser scan sensor as Fig. 6 shows. Usually, there are about 7 cycles per second when laser scan sensor is rotating, whose outputted format is two-dimensional array data. One dimension is the angle value of the obstacles ranging from 1 to 360 degrees, and the other dimension is the distance of the obstacles which is measured on each angle value.

Comparation between the angle of rotation α which is from the outputted data of ROS topic and the angle of rotation α which is from the laser scan sensor, if the difference of them is less than 5%, they can be averaged to eliminate errors, otherwise, the robot needs to rotate back to the fridge, then repeating the action that robot rotates from fridge to the washing machine again to eliminate the error from unexpected situation. In the above calculation, if the angle γ crosses 360 degrees, α = γ + 360-β.

**Fig. 7:**
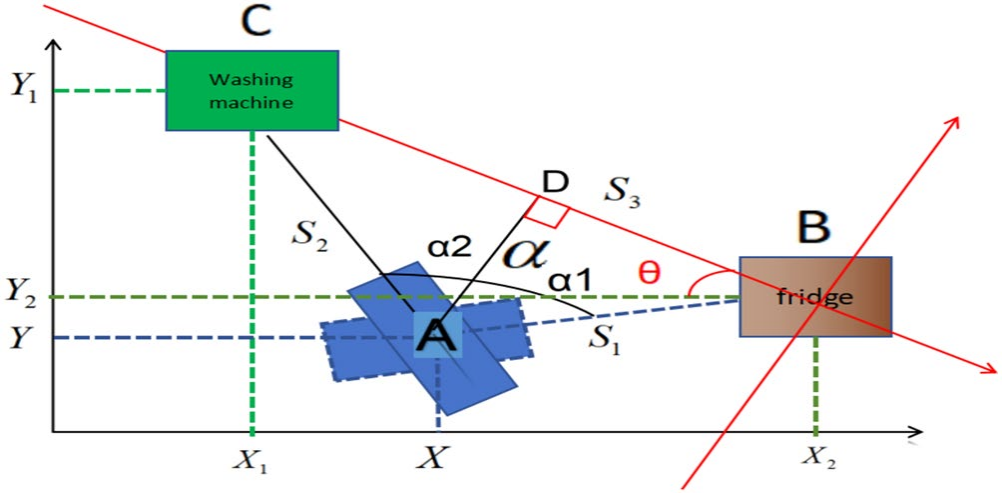
Calculation of the location of the robot after translation and rotation of coordinate axes.

In addition to the angle of rotation α can be calculated in Fig. 2, the distance S1 between the robot and the fridge which is line AB , and the distance S2 between the robot and the washing machine which is line AC in Fig. 2, both of them can also be calculated from the laser scan sensor, as shown in Fig. 7.

The distance and angle values of each obstacle can be outputted from laser scan sensors, which contains noise data that needs to be eliminated by clearing outliers algorithm. In Fig. 7, when the home service robot is at the position of blue dashed rectangle which orientates to the fridge, the average distance is S1, and when the home service robot is in the position of blue solid line which orientates to the washing machine, the average distance is S2, so the value of S1 and S2 can be taken from 350–10 angle degrees (350–360 degrees and 1–10 degrees) per cycle, because laser scan sensor rotates 7 cycles in one second, so the value of S1 and S2 should be averaged in 7 cycles in one second.

#### The localization of position of home service robot with least power consumption and result analysis

The angle of rotation α and distance S1 in Fig. 7 can be obtained according to above analysis, in order to obtain the location of robot A in Fig. 7 simply, the coordinate axes should be rotated as shown in Fig. 7, and the red lines are the new axis after rotation, the new red X axis is the line which connects the center point of fridge and washing machine, the new origin of coordinate is the position of fridge B.

The coordinate value of the position of fridge B in the new axis is (0,0), when α and distance S1 are determined, the value of distance S2 can be obtained from the outputted data of the laser sensor, the length of distance S3 which is the distance between fridge and washing machine can be calculated according to the cosine law. The vertical line from position of robot A to X axis in new red coordinate axes are intersect at point D, because the angle of rotation α and the distance S1 and S2 can be obtained, and the coordinate value of point C can be calculated according to formula (1), which is (-$\left( {{\mathbf{S}}_{{\mathbf{1}}}^{{\mathbf{2}}} + {\mathbf{S}}_{{\mathbf{2}}}^{{\mathbf{2}}} - {\mathbf{2}}\cos {{\varvec{\upalpha}}} \times {\mathbf{S}}_{{\mathbf{1}}} {\mathbf{S}}_{{\mathbf{2}}} } \right)^{{{\mathbf{1}}/{\mathbf{2}}}}$, 0).

1 $${\text{S}}_{{3}} = {(}S_{1}^{2} + S_{2}^{2} - {\text{2cos}}\alpha \times S_{1} S_{2} )^{1/2}$$

Starting from robot’s point A, drawing a straight line perpendicular to the new X axis and divide angle α into two parts, one being α1 and the other α2 as shown in Fig. 7, the equation can be obtained in formula (2).

2 $$S_{1} {\text{cos}}\alpha_{1} = S_{2} \cos (\alpha - \alpha_{1} ) = S_{2} ({\text{cos}}\alpha \cos \alpha_{1} + \sin \alpha \sin \alpha_{1} )$$

The deformation of formula (2) is

3 $$(S_{1} - S_{2} {\text{cos}}\alpha )\cos \alpha_{1} = \sin \alpha \sin \alpha_{1}$$

Square the both sides

4 $$(S_{1} - S_{2} {\text{cos}}\alpha )^{2} \cos^{2} \alpha_{1} = \sin^{2} \alpha (1 - \cos^{2} \alpha_{1} )$$

From formula (4), it is can be obtained:

5 $${\text{cos}}\alpha_{1} = \frac{\sin \alpha }{{\sqrt {(S_{1} - S_{2} \cos \alpha )^{2} + \sin^{2} } \alpha }}$$

6 $${\text{sin}}\alpha_{1} = \frac{{S_{1} - S_{2} \cos \alpha }}{{\sqrt {(S_{1} - S_{2} \cos \alpha )^{2} + \sin^{2} } \alpha }}$$

So the coordinate value of position of robot A (A1x, A1y) is ($S_{1} \frac{\sin \alpha }{{\sqrt {(S_{1} - S_{2} \cos \alpha )^{2} + \sin^{2} } \alpha }}$, $S_{1} \frac{{S_{1} - S_{2} \cos \alpha }}{{\sqrt {(S_{1} - S_{2} \cos \alpha )^{2} + \sin^{2} } \alpha }}$) which is the position of the robot in the new coordinate axes in Fig. 7.

If returning to the original coordinate axes, the new coordinate axes should first be translated to the coordinate value first, then rotating to angle θ. So the new coordinate value of the point A (A2x, A2y) can be expressed as (A1x + X2, A1y + Y2).

The formula (7) and (8) can be obtained from the Fig. 7,

7 $$\sin \theta = \frac{{Y_{1} - Y_{2} }}{{S{}_{3}}} = \frac{{Y_{1} - Y_{2} }}{{{(}S_{1}^{2} + S_{2}^{2} - {\text{2cos}}\alpha \times S_{1} S_{2} )^{1/2} }}$$

8 $${\text{cos}}\theta = \frac{{X_{2} - X_{1} }}{{S{}_{3}}} = \frac{{X_{2} - X_{1} }}{{{(}S_{1}^{2} + S_{2}^{2} - {\text{2cos}}\alpha \times S_{1} S_{2} )^{1/2} }}$$

Then the axis will rotate the angle -θ, the new coordinate value of point A is (A3x, A3y), the relationship between (A2x, A2y) and (A3x,A3y) is formula (9)

9 $$\begin{gathered} A2x = A3x\cos ( - \theta ) - A3y\sin ( - \theta ) \hfill \\ A2y = A3x\sin ( - \theta ) - A3y\cos ( - \theta ) \hfill \\ \end{gathered}$$

Based on the above analysis, the formula (10) can be obtained

10 $$\begin{gathered} S_{1} \frac{\sin \alpha }{{\sqrt {(S_{1} - S_{2} \cos \alpha )^{2} + \sin^{2} } \alpha }} + X_{2} = A3x\frac{{X_{2} - X_{1} }}{{{(}S_{1}^{2} + S_{2}^{2} - {\text{2cos}}\alpha \times S_{1} S_{2} )^{1/2} }} + A3y\frac{{Y_{1} - Y_{2} }}{{{(}S_{1}^{2} + S_{2}^{2} - {\text{2cos}}\alpha \times S_{1} S_{2} )^{1/2} }} \hfill \\ S_{1} \frac{{S_{1} - S_{2} \cos \alpha }}{{\sqrt {(S_{1} - S_{2} \cos \alpha )^{2} + \sin^{2} } \alpha }} + Y_{2} = - A3x\frac{{Y_{1} - Y_{2} }}{{{(}S_{1}^{2} + S_{2}^{2} - {\text{2cos}}\alpha \times S_{1} S_{2} )^{1/2} }} - A3y\frac{{X_{2} - X_{1} }}{{{(}S_{1}^{2} + S_{2}^{2} - {\text{2cos}}\alpha \times S_{1} S_{2} )^{1/2} }} \hfill \\ \end{gathered}$$

The coordinate values of the point A (A3x, A3y) can be expressed by the formula (11) after the translation and rotation of the axis.

11 $$\begin{gathered} A3{\text{x}} = \frac{{{(}S_{1}^{2} + S_{2}^{2} - {\text{2cos}}\alpha \times S_{1} S_{2} )^{1/2} }}{{(X_{2} - X_{1} )^{2} (Y_{1} - Y_{2} )^{2} }} \times \left[ {(X_{2} - X_{1} )(S_{1} \frac{\sin \alpha }{{\sqrt {(S_{1} - S_{2} \cos \alpha )^{2} + \sin^{2} } \alpha }} + X_{2} ) + (Y_{1} - Y_{2} )S_{1} \frac{{S_{1} - S_{2} \cos \alpha }}{{\sqrt {(S_{1} - S_{2} \cos \alpha )^{2} + \sin^{2} } \alpha }} + Y_{2} } \right] \hfill \\ A3y = \frac{{{(}S_{1}^{2} + S_{2}^{2} - {\text{2cos}}\alpha \times S_{1} S_{2} )^{1/2} }}{{(X_{2} - X_{1} )^{2} (Y_{1} - Y_{2} )^{2} }} \times \left[ {(Y_{1} - Y_{2} )(S_{1} \frac{\sin \alpha }{{\sqrt {(S_{1} - S_{2} \cos \alpha )^{2} + \sin^{2} } \alpha }} + X_{2} ) + (X_{2} - X_{1} )S_{1} \frac{{S_{1} - S_{2} \cos \alpha }}{{\sqrt {(S_{1} - S_{2} \cos \alpha )^{2} + \sin^{2} } \alpha }} + Y_{2} } \right] \hfill \\ \end{gathered}$$

Because the coordinate values of (X1, Y1) and (X2, Y2) are known, the angle of rotation α, as well as distance S1 and S2 can be outputted from the laser scan sensor, so the location of the robot is the coordinate value of the point A (A3x, A3y) can be obtained by the formula (11).

If using the matrix transformation for position transformation, the whole process can be separated into two steps, the first is the transformation of the axis, the second is the rotation of the axis, the above formulas can be expressed as formula (12).

12 $$\left[ {\begin{array}{*{20}c} {X_{A3} } \\ {Y_{A3} } \\ 1 \\ \end{array} } \right] = \left[ {\begin{array}{*{20}c} 1 & 0 & {T{\text{x}}} \\ 0 & 1 & {Ty} \\ 0 & 0 & 1 \\ \end{array} } \right]\left[ {\begin{array}{*{20}c} {\cos \theta } & { - \sin \theta } & 0 \\ {\sin \theta } & {\cos \theta } & 0 \\ 0 & 0 & 1 \\ \end{array} } \right]\left[ {\begin{array}{*{20}c} {X_{A} } \\ {Y_{A} } \\ 1 \\ \end{array} } \right]$$

According to the above analysis, in formula (12) $Tx = S_{1} \frac{\sin \alpha }{{\sqrt {(S_{1} - S_{2} \cos \alpha )^{2} + \sin^{2} } \alpha }}$, $T{\text{y}} = S_{1} \frac{{S_{1} - S_{2} \cos \alpha }}{{\sqrt {(S_{1} - S_{2} \cos \alpha )^{2} + \sin^{2} } \alpha }}$ , cosθ and sinθ can be obtained from formula (7) and (8).

The calculation of SLAM is very complex that contains the Singular Value Decomposition (SVD), point cloud, Jacobian matrix and Hessian matrix, multivariate Gaussian distribution, Extended Kalman Filter, Particle Filter, over determined system of equations, quaternion, large-scale graph optimization, Lie groups and Lie algebras, which relates with complicated mathematical theory and calculation, this calculation should be run continuously in SLAM, which consumes lots of CPU resources, as the power of CPU takes the main part of power of the whole system, when SLAM algorithm is running in mobile robot, it will raise the power consumption of the mobile robot significantly.

This method simplifies calculation in SLAM, only the calculation of matrix as formula (12) shows, compared with SLAM algorithm, it reduces much work load of CPU, then reducing the power consumption of CPU and saving the power consumption of battery.

### The localization of angle with least power consumption

Usually there are two types localization of robot, one is localization of position which has been discussed in the Section "The localization of position of home service robot with least power consumption and result analysis.", the other is localization of angle which will be discussed in the following section.

Many mobile robots are working in the indoor environment where needs IMU or MEMS sensors for the localization of angle, but IMU sensor or MEMS sensor has much error when working in the indoor environment. Many solutions have been proposed to reduce the error, such as wifi, RFID localization, or SLAM technology which can help the robot to obtain more accurate localization of angle, but they will increase the cost of the devices and lead more power consumption, for example, for wifi locating, it needs to equip a wifi module on the robot and a base station in the environment. For RFID locating, the RFID module should be added on the robot and many RFID tags should be pasted to the ground before starts working; for SLAM technology, the map of the house should be built first, and a large number of data feature extraction and data point matching algorithms should be processed frequently, which increases the load of the CPU, thus increasing the power consumption of the robot, when the home appliance and furniture are moved, added or removed in the house, the map needs to be built again.

#### The judgment of localization of angle with the least power consumption

There is another method that can predict accurate angle in the indoor environment, usually there is a motor inside the wheel of robot, photoelectric encoder is placed behind the motor to calculate how many circles the wheel rotates. When the angle of rotation of wheel is 2π, the pulse amount of the photoelectric encoder is M, when the wheel angle of rotation is θ, the pulse amount of the photoelectric encoder is m, then the wheel angle of rotation is as follows:

13 $$\theta = {2}\pi {\text{m}}/{\text{M}}$$

**Fig. 8:**
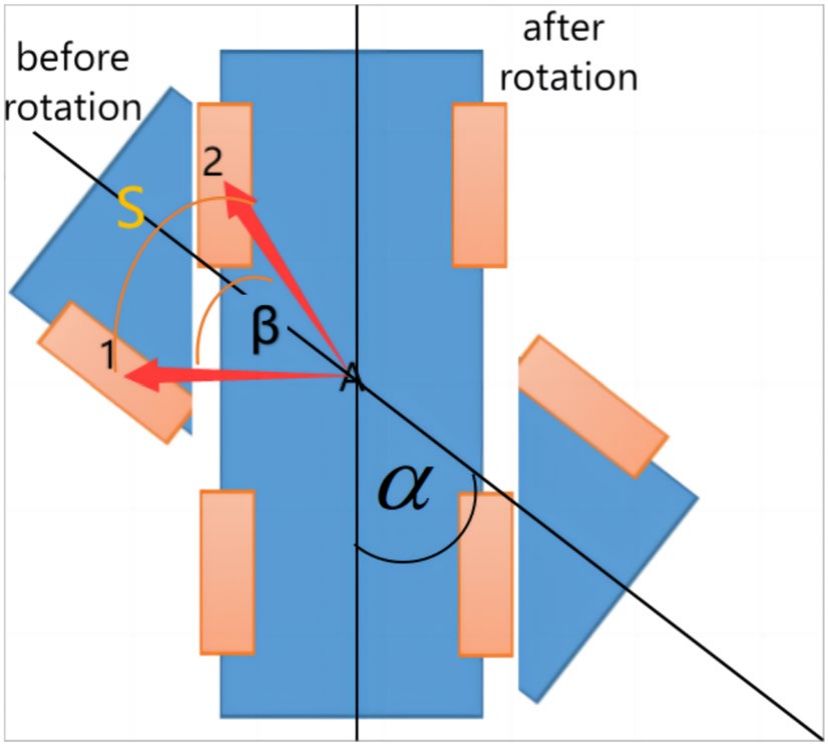
Calculation model of robot rotation.

In Fig. 8, the blue rectangle is the robot, and the orange rectangle is the wheel of the robot. The whole robot is a rigid body, the distance between the wheel and the main body will not be changed in the movement, and the angle of rotation β of the wheel is the same as that of the robot α in Fig. 8, so β = α, and β can be counted according to θ in formula (13).

In Fig. 8, A is the center of gravity of the robot, A is on the center of the base if the robot is bilateral symmetry. Before rotation, the center of the top left wheel is marked as 1, after rotation, it is marked as 2, the distance traveled by the wheel is the curve length S, so the value of S is S = βk in Fig. 8. If the radius of the wheel is d, then the value of s is s = θd, so βk = θd, and β can be calculated according to formula (14).

14 $$\alpha = \beta = \theta {\text{d}}/{\text{k}} = {2}\pi {\text{md}}/{\text{Mk}}$$

In formula (14), m, d, k can be measured on the robot. According to formula (13), M can be obtained from the user’s manual of the motor, so the angle of rotation α of the robot can be obtained according to formula (14).

**Fig. 9:**
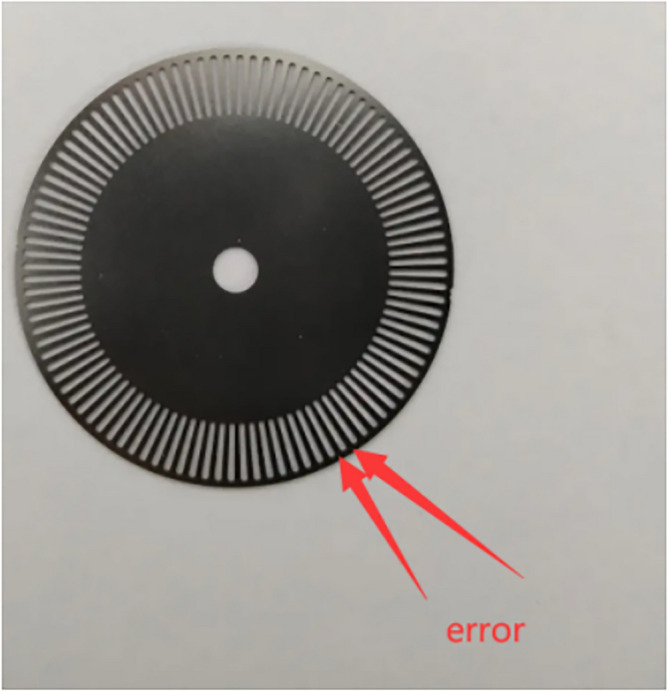
Grating of photoelectric encoder.

The above calculation is an ideal model without considering the error from devices and environment. As shown in Fig. 9, there are errors in every gap of the grating of the photoelectric encoder, if there are 100 gaps in the grating, and the width of the black shield and the width of the gap are the same, the maximum error is 360/ (100*2) = 1.8 degrees.

Usually it is random for the moment when the robot stops rotating, so the error from the grating obeys the uniform distribution. The minimum error is 0, the maximum error is 1.8 degrees, then the average error is 0.9 degrees, so the α in the formula (14) needs to be corrected, and the corrected α can be obtained from the formula (15).

15 $$\alpha = {2}\pi {\text{md}}/{\text{Mk}} + 0.{9}*{2}\pi /{36}0$$

In addition to the error from the grating, there is another error from the ground friction, the wheel may slip when it moves on the ground, especially when it moves on smooth ground, this error is a accumulative error, the robot continues moving, the accumulative error will become larger and larger.

**Fig. 10:**
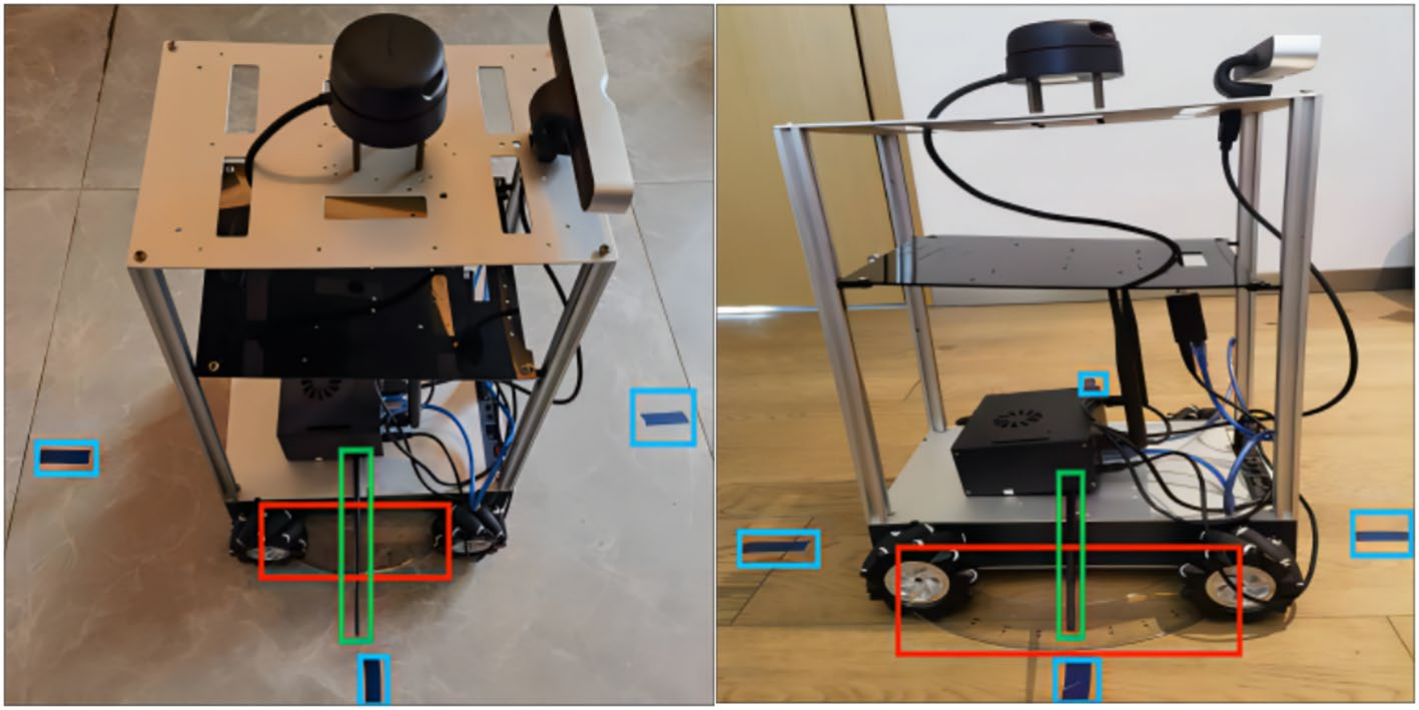
Robot moves on the ground with different friction forces.

In order to measure the wheel slip error from different friction forces on different surfaces, gluing a large circular ruler on the ground and place it under the robot as the red rectangle shows in Fig. 10, fixing the long dark line on the robot, and its end is outside of the robot as the green rectangle shows. The robot will rotate angle α until α reaches 90 degrees, then check how much degree the end of the long dark line points. This process can be tested on different surfaces to obtain the test error, so the formula (15) should also add the error *f* that is from the friction force, which can be modified to the formula (16).

16 $$\alpha = 2\pi md/Mk + 0.9*2\pi /360 + f$$

#### The accurate localization of angle is predicted by machine learning algorithms and result analysis

Machine learning algorithms can predict the localization of angle in the indoor environment more accurately, the data set contains two properties, one property is the angle data of the MEMS module, the other property is the angle data α in formula (16) which comes from the odometer data. The true value of localization of angle can be measured from the circular ruler which is mentioned above. Training data accounted for 70% of the total data set, and test data accounted for 30% of the total data set.

The first machine learning algorithm is the decision tree algorithm, each node in the tree represents the test of the feature, the branch of the tree represents each test result of the feature, and each leaf node of the tree represents the category. The API is decisiontreecclassifier from sklearn. Parameters are listed following: the value of criterion is “CART”, the value of splitter is “best”, the value of max features is the default value “None”, that is the default number of features in the original data set, the value of max depth is “None”, and there is no depth limit. The value of max leaf nodes is the default value “None”, and there are no leaf node restrictions.

**Fig. 11:**
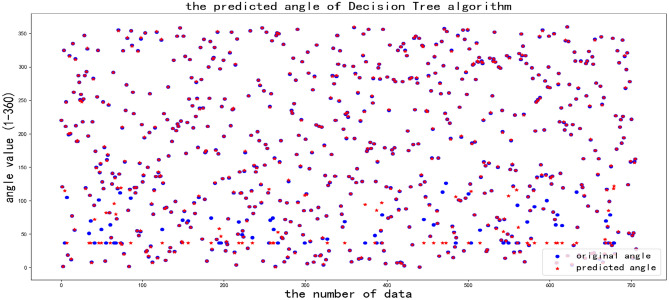
Analyzing the original angle and predicted angle with Decision Tree Algorithm.

In Fig. 11, there is a phenomenon that some of the raw data differs from the predicted data when the angle is less than 120 degrees, but it occurs rarely when the angle is larger than 120 degrees, after many tests, the accuracy rate approaches to 89%.

The second machine learning algorithm is linear support vector machine regression (linear SVR), which is characterized by tolerating the maximum error between the model output and the true value, and calculating the loss value only if the error exceeds the width between the model output and the true value.

The API is LinearSVR () from the sklearn.svm package with the following parameters, epsilon is “0”, loss is “epsilon_insensitive”, and intercept_scaling is “1.0”.

**Fig. 12:**
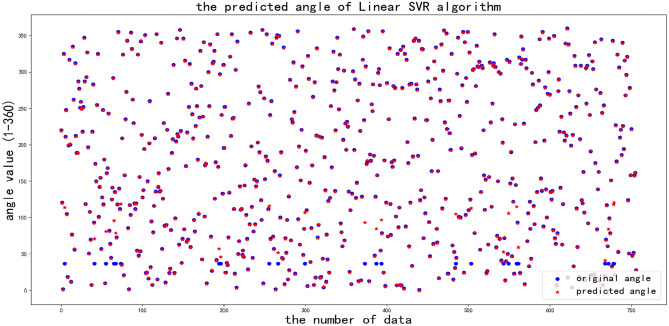
Raw angle and predicted angle obtained by linear SVR algorithm.

As shown in Fig. 12, when the original angle is about 40 degrees, most of the predicted angle is different from the original angle, and when the original angle is other angles, most of the predicted angle is the same as the original angle. After many tests, the accuracy rate can reach about 96%.

The third machine learning algorithm is the Naive Bayes method, which is proposed based on the Bayes principle and uses probabilistic statistical knowledge to classify sample datasets.

**Fig. 13:**
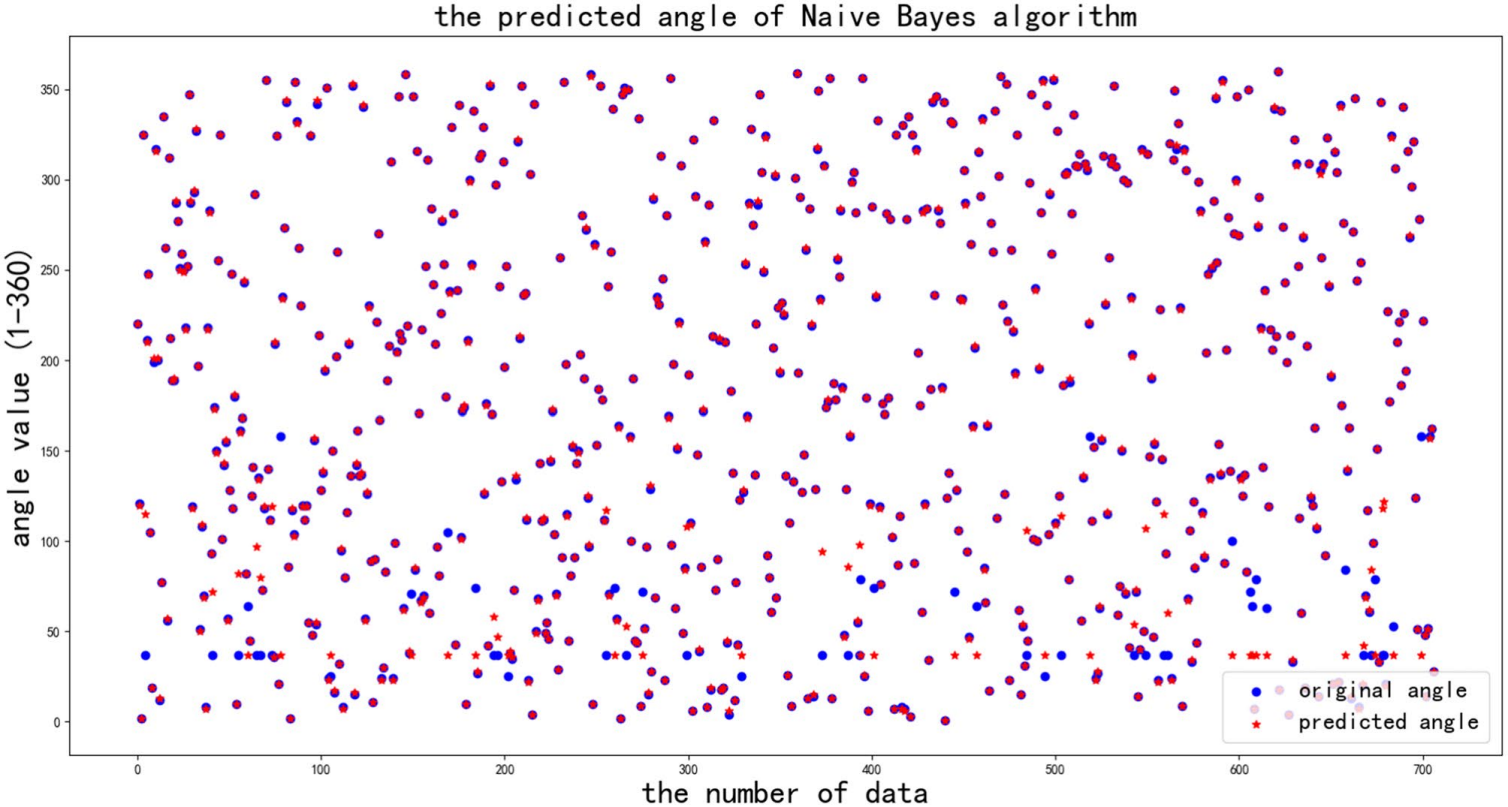
Raw and predicted angles obtained from Naive Bayes algorithm.

The API is GaussianNB () from the sklearn.naive_bayes package, the parameters are default. In Fig. 13, when the angle is larger than 20 degrees while less than 120 degrees, some of the original data is different from the predicted data, and when the angle is in other scopes, this phenomenon is relatively rare, which is the similar as the decision tree algorithm, the accuracy rate is about 91%.

From the results of the above three machine learning algorithms, it can be seen that when the angle value is less than 80 degrees, more blue dots don’t match the red dots and this error is obvious, this phenomena will be analyzed from the two property data in the machine learning algorithms, which is angle outputted from MEMS sensor and odometer data.

Because the home service robot works in indoor environment, and the testing environment is also indoors. The output angles from MEMS sensors have a relatively large error in the indoor environment . When the robot rotates a small angle, the changes in the Earth’s magnetic field is not significant at this time, the error from uncertainty of the MEMS sensors at this time will take a relatively large portion of the whole error. For the same reason, when the robot rotates a small angle, the error of odometer data shows in Fig. 9 will take a relatively large portion of the whole error. So the uncertainty of the predicted angle become obvious when the robot rotates a small angle.

It can concluded that the linear SVR algorithm is more accurate than the other two algorithms, and the more accurate localization of angle can be predicted by the linear SVR algorithm.

After the localization of angle is obtained, the moving distance of the robot can be obtained as following, when the robot moves forward, the distance can be calculated according to formula (17).

17 $$S = 2\pi md/M + (0.9 \times 2\pi /360 + {\text{f)}} \times {\text{k}}$$

If the robot moves backwards, the distance can be calculated according to formula (18).

18 $$S = - 2\pi md/M - (0.9 \times 2\pi /360 + {\text{f)}} \times {\text{k}}$$

## Path planning with the least power consumption

Path planning is important to the power consumption of the robot, robot needs to move on the designed path continuously, different path leads to different moving distance and rotating angle, which leads to different power consumption in the movement, so planning a path which can save the power consumption is important to the robot.

### Supplement and optimization of the previous path planning algorithms

In the previous path planning algorithms, the whole power consumption values are trained in different machine learning models. This paper proposes a new method, that the power consumption values is computed which only come from the motion of robot, and the power values consumption needs to be deleted from the database which are from the other devices.

#### The power consumption that only comes from the movement of robot

First, the power consumption of the robot in the stopped state can be recorded as A, which represents the power consumption of CPU, RAM, camera, laser scan sensor and other devices, but not contains the power consumption of the movement of robot. Then, the power consumption when the robot is moving can be recorded as B, which is the whole power consumption that contains the power consumption of all devices and movement of the robot, so the power consumption only for movement of robot can be record as C, that C = B-A. The following is how to obtain the power consumption C from the data in the database (Table 4).

**Table 4:** The data table of values from /battery topic in one second.

Second	Voltage	Current
1,691,289,815	12.17399979	0.312000006
1,691,289,816	12.17399979	0.1949999928469999
1,691,289,817	12.17399979	0.1949999928469999
1,691,289,818	12.20800018	0.200000003
1,691,289,819	12.19799995	0.611999989
1,691,289,820	12.19799995	0.279000014
1,691,289,821	12.16499996	0.412
1,691,289,822	12.13599968	0.400000006
1,691,289,823	12.09399986	0.508000016
1,691,289,824	12.05599976	0.158000007
1,691,289,825	12.116999626199998	0.187000006
1,691,289,826	12.15499973	0.0289999991655

In Table 5, from the variance values of voltage and current, it is obvious that in the process of movement of robot, the current changes dramatically, while voltage changes rarely and keep about 11 V, because the robot is powered by 12 V battery, which can output large current when robot moves fast at some moment, while the value of voltage will not drop rapidly and keep about 11 V at this moment. It is the 95% confidence intervals in Table 5, for the values in row “margin_of_error/mean” in Table 5, because of the same reason, the changes of values in current is more dramatically than changes of values of voltage.

**Table 5:** The statistical values of data in Table 4.

	Second	Voltage	Current
Mean	1,691,290,798.1923077	11.7762040920353	0.5448709676179682
Variance	292,119.15532544453	0.031108777308371166	0.17419822317352363
Std_deviation	540.4804856102064	0.17637680490464488	0.41737060650400815
Confidence_interVal_0.025	1,691,290,771.7799757	11.767584867366216	0.5244747991440283
Confidence_interVal_0.975	1,691,290,824.6046398	11.784823316704385	0.565267136091908
Margin_of_error/mean	1.561667104499453e− 08	0.0007319187576678131	0.037433024855602945

The voltage and current value in each second can be outputted from /battery topic in ROS, and a new key “power” is added in the merged data table whose value is the multiplication of voltage and current as Table 4 shows. Tables 3 and 4 can be merged with the SQL command “groupby” with primary key “second” in the database as Table 4 shows.

In Table 6, the key lx, ly and lz represent the linear velocity of the robot in the three directions of rectangular coordinate system x–y-z; the key ax, ay, az represent the robot’s angular velocity in the three directions of rectangular coordinate system x–y-z, according to the above C = B-A, the average power consumption value A can be calculated when lx, ly, lz, ax, ay, az are all 0 in Table 6, then the power consumption C can be easily obtained.

**Table 6:** All the data in one second.

Second	px	py	pz	ox	oy	oz	ow	lx
1,691,289,815	− 0.064948742	− 0.409979159	0	0	0	− 0.809016994	0.587785252	0
1,691,289,816	− 0.064948742	− 0.409979159	0	0	0	− 0.809016994	0.587785252	0
1,691,289,817	− 0.064949722	− 0.409982176	0	0	0	− 0.809016994	0.587785252	0.00016
1,691,289,818	− 0.115638127	− 0.564948065	0	0	0	− 0.810750493	0.58537727145446	0.28578
1,691,289,819	− 0.158213113	− 0.68890615	0	0	0	− 0.808810589	0.5880667536703599	0.021879999999999997
1,691,289,820	− 0.143509438	− 0.627549311	0	0	0	− 0.786686837	0.61706287098312	− 0.16102
1,691,289,821	− 0.114134145	− 0.455068063	0	0	0	− 0.744415851	0.6674572394846401	− 0.19116
1,691,289,822	− 0.106861958	− 0.216829638	0	0	0	− 0.697028122	0.7167697696054001	− 0.27274
1,691,289,823	− 0.126755151	0.02187789802805419	0	0	0	− 0.651639977	0.75840469445016	− 0.14368
1,691,289,824	− 0.131185039	0.052635177314834004	0	0	0	− 0.645835545	0.76343224764978	0.07314
1,691,289,825	− 0.101356816	− 0.234587799	0	0	0	− 0.691455116	0.72204993982858	0.29552
1,691,289,826	− 0.105215073	− 0.470436822	0	0	0	− 0.724807927	0.6887789033661601	0.45549999999999996
1,691,289,827	− 0.166707348	− 1.120029436	0	0	0	− 0.753941042	0.65677830556908	0.708
1,691,289,828	− 0.297630241	− 1.81669801	0	0	0	− 0.787597884	0.61600315180228	0.7101000000000001

Second	ly	lz	ax	ay	az	Voltage	Current	Power
1,691,289,815	0	0	0	0	0	12.17399979	0.312000006	3.798288011720836
1,691,289,816	0	0	0	0	0	12.17399979	0.1949999928469999	2.373929871267378
1,691,289,817	0	0	0	0	8e− 05	12.17399979	0.1949999928469999	2.373929871267378
1,691,289,818	8e− 05	0	0	0	− 0.00088	12.20800018	0.200000003	2.4416000729998406
1,691,289,819	0.0008	0	0	0	0.010959999999999994	12.19799995	0.611999989	7.465175832376489
1,691,289,820	− 0.00088	0	0	0	0.12041999999999999	12.19799995	0.279000014	3.4032421588110653
1,691,289,821	− 0.00064	0	0	0	0.11963999999999998	12.16499996	0.412	5.011979990105504
1,691,289,822	0.00024	0	0	0	0.12289999999999997	12.13599968	0.400000006	4.854399944170558
1,691,289,823	0.00016	0	0	0	0.06581999999999998	12.09399986	0.508000016	6.143752126319526
1,691,289,824	0.0010400000000000001	0	0	0	− 0.05536	12.05599976	0.158000007	1.9048480491034303
1,691,289,825	− 0.00056	0	0	0	− 0.12578	12.116999626199998	0.187000006	2.265879008096526
1,691,289,826	0.0030399999999999997	0	0	0	− 0.08218	12.15499973	0.0289999991655	0.35249498211365277
1,691,289,827	0.0036	0	0	0	− 0.0805	12.05599976	2.275000095	27.427400594453196
1,691,289,828	0.00432	0	0	0	− 0.07926	11.9989996	0.8870000243189999	10.643112933100872

**Fig. 14:**
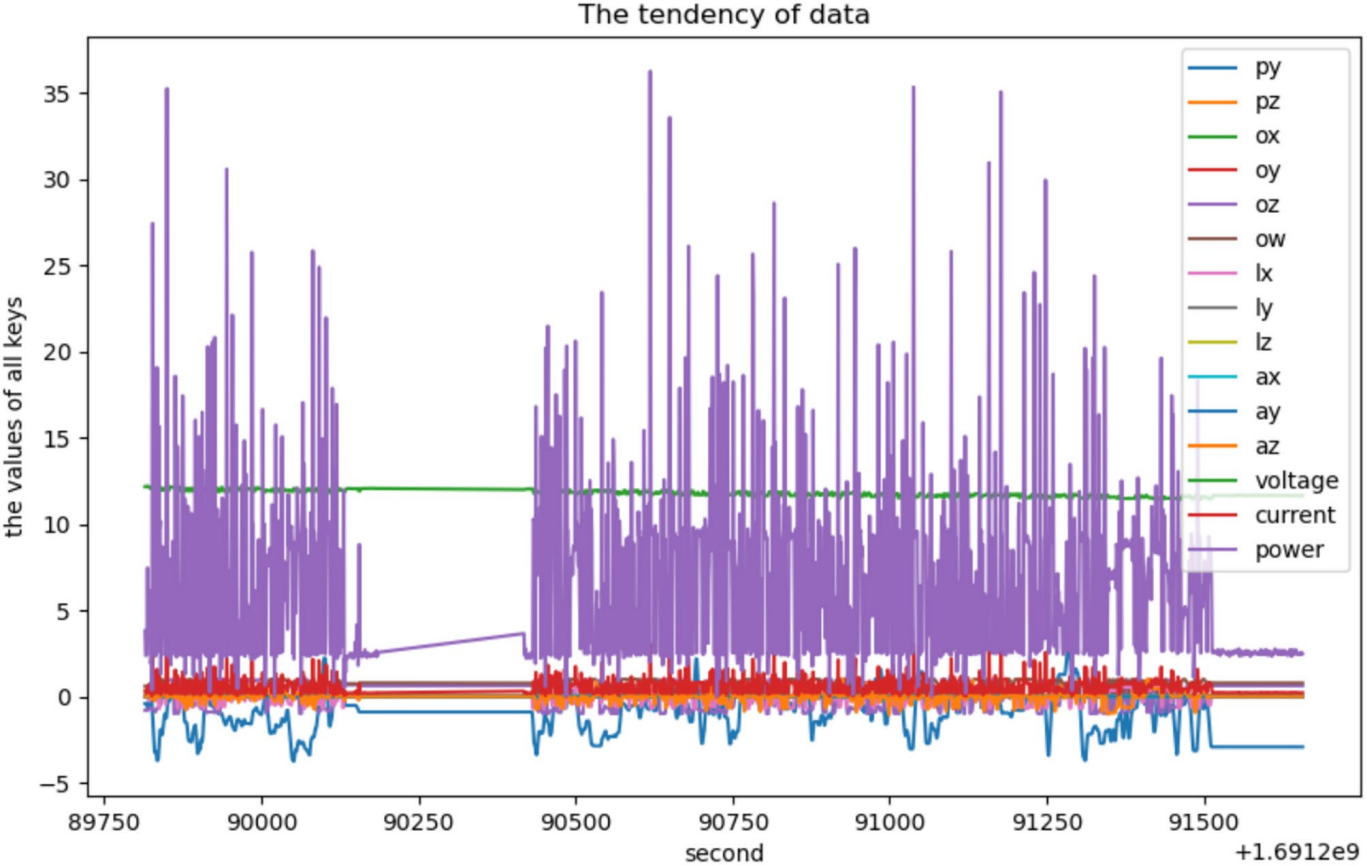
The tendency of data in Table 6.

All the data in Table 6 are changing with time and the tendency can be shown in Fig. 14, the purple color line represents the power consumption, the robot stops movement from 1,691,290,157 s to 1,691,290,430 s, and the purple line keeps a straight line during this period, the reason that the robot stops movement in this period is to get the average power consumption value when robot stops, which can be used to calculate the power consumption only for the movement of the robot, this has been explained in Sect. 3.1.3.

From the keys of Table 7, the following can be obtained, from the comparison of px and py, the changes of horizontal position is more dramatically than changes of vertical position because the robot moves longer distance on horizontal direction, but most of the time the robot moves around the mean position, rarely moves far away the mean position in the test.

**Table 7:** The statistical values of data in Table 6.

	px	py	pz	ox	oy	oz	ow	lx
Mean	− 1.535987694	− 1.047626707	0	0	0	0.10322047604375187	0.6282677954040058	− 0.008057834
Variance	3.623633005405085	1.5150362728162912	0	0	0	0.4652816431475158	0.09760541456689871	0.12410324829552889
Std_deviation	1.903584252247608	1.2308680972453105	0	0	0	0.682115564363925	0.3124186527192298	0.3522829094570568
Confidence_interval_0.025	− 1.629012513	− 1.107777069	0	0	0	0.0698866864748174	0.6130004445748776	− 0.025273281
Confidence_interval_0.975	− 1.442962875	− 0.987476345	0	0	0	0.13655426561268635	0.643535146233134	0.009157613250088067
Margin_of_error/mean	− 0.060563518	− 0.057415835				0.32293776241455563	0.02430070575129604	− 2.136485747

	ly	lz	ax	ay	az	Voltage	Current	Power
Mean	0.00019606136251026468	0	0	0	− 0.016508854	11.7762040920353	0.5448709676179682	6.411329527275272
Variance	4.421391021983007e− 06	0	0	0	0.08573606333536214	0.031108777308371166	0.17419822317352363	24.27496247068035
Std_deviation	0.0021027103989810407	0	0	0	0.29280721189096787	0.17637680490464488	0.41737060650400815	4.926962803866125
Confidence_interval_0.025	9.330559849334969e− 05	0	0	0	− 0.030817828	11.767584867366216	0.5244747991440283	6.170557508722682
Confidence_interval_0.975	0.0002988171265271797	0	0	0	− 0.00219988	11.784823316704385	0.565267136091908	6.652101545827861
Margin_of_error/mean	0.5241000200206977				− 0.866745454	0.0007319187576678131	0.037433024855602945	0.03755414809491379

From the comparison of px, py and oz, the changes of angle is more dramatically than linear movement because the robot usually rotates large angle in place or move on high curvature curve path in the experiment, sometimes the robot can rotate even 360 degrees or more to finish some tasks. And most of time the robot rotates large angles, but not move far away from the initial position in the test.

From the comparison of lx and ly, the changes of vertical velocity is more dramatically than changes of horizontal velocity because there is less obstacles on the vertical direction, most of the time the vertical velocity of robot is near the average vertical velocity, and sometimes the horizontal velocity of robot is less than the average horizontal velocity.

From the comparison of lx, ly and az, the changes of angular velocity is more dramatically than linear velocity because sometimes the robot move on high curvature curve path or rotate in place, the components of speed in the horizontal and vertical directions are relatively less than those in the angular direction, most of the time the vertical velocity of robot is near the average vertical velocity, and the horizontal velocity and angular velocity of robot is less than the average velocity.

From the comparison of voltage and current, the analysis has been conducted in the above part, the changes of current is more dramatically than voltage because sometimes the robot moves fast and current will changes more, but the voltage will be stable around 11 V because the robot is powered by 12 V battery, most of the time the voltage is near the average voltage, and the current is more than the average current, because the power is mostly determined by the current, sometimes the power is more than the average power.

#### The processing of noise data in the databases

In the previous algorithms, abnormal data is picked out with box plot algorithm, but noise data is not picked. In the new method, noise data is picked and deleted.

Because the time interval between each row is 1 s in Table 6, if a value in a row is greater than 3 times of the value of the same key in the previous row and the next row, or a value in a row is less than 1/3 times of the value of the same key in the previous row and the next row, the value is considered as noise data, the entire row should be deleted in the database.

### The experiment and theoretical analysis of power consumption for the path planning with least power consumption and result analysis

According to the law of energy conservation, P = F*S can be obtained, F is the driving force of the robot, S is the moving distance on the moving path, and P only represents the power consumption generated from the movement of robot. According to Newton’s second law, F = ma, m is the mass of the robot, and a is the acceleration of the movement of robot. Because acceleration represents the changes in velocity, so a = ∆$\text{v}/\Delta \text{t}$=dv. Through the above analysis, the following formula can be obtained:

19 $$P = m\sum\limits_{i = 0}^{n} {dv_{i} \times dS_{i} }$$

In formula (19), d is the differential sign, because the data can be outputted from /odom topic for about 50 times per second, on average it is every 20 ms for once, so dt is 20 ms, P in formula (19) only relates with dv and dS.

In the /odom topic, the distance of the moving path is ox,oy,oz,ow,px,py,pz, where the fields ox,oy,oz,ow are quaternions, representing the angle value of the rotation of the robot, and px,py,pz represent the distance of the robot moving in the three directions of rectangular coordinate system x–y-z.

Usually the moving trajectory of home service robot is curve, sometimes the moving trajectory is straight path, so moving distance S is the distance along this curve or straight path. When dt is about 20 ms, dS can be taken as the moving distance in the time range of dt.

**Fig. 15:**
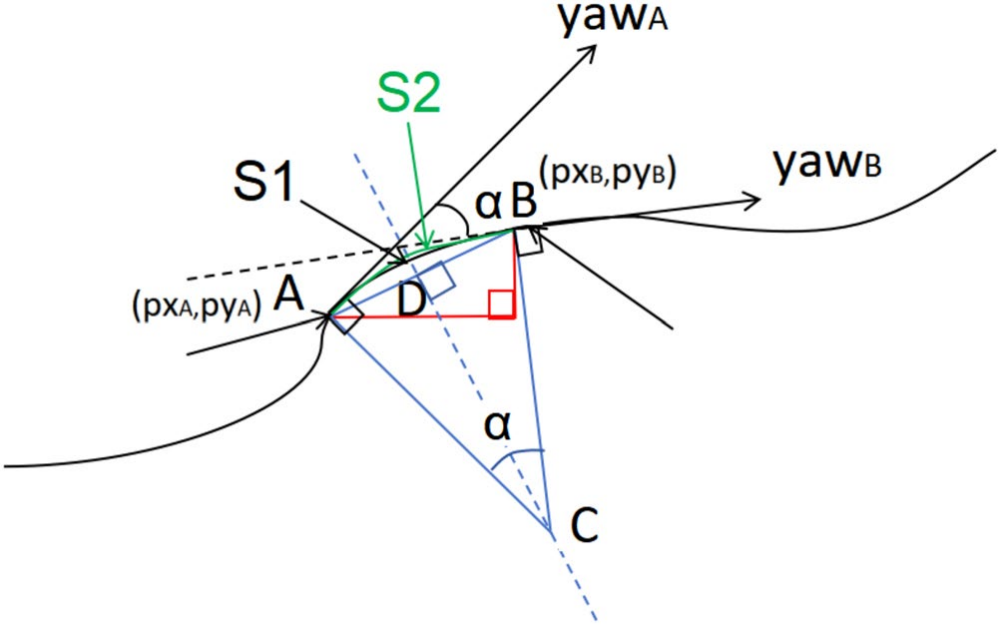
Calculation of distance of robot moving curve.

As shown in Fig. 15, the robot’s moving path is a black curve, dt is 20 ms, in the dt, the robot moves from point A along the black curve to point B and the moving distance is S1. The direction of the robot at point A is ${\text{yaw}}_{\text{A}}$, which points to the tangential direction of point A, and the direction of the robot at point B ${\text{yaw}}_{\text{B}}$ points to the tangential direction of point B, and ${\text{yaw}}_{\text{A}}$ and ${\text{yaw}}_{\text{B}}$ are all radian values. The normal of the robot at point A and the normal of the robot at point B intersect at point C, then AC is perpendicular to ${\text{yaw}}_{\text{A}}$, and BC is perpendicular to ${\text{yaw}}_{\text{B}}$.

The values of the output quaternions ox, oy, oz, ow in /odom are recorded as x,y,z,w respectively, then the orientation angle of the robot that the value of yaw can be expressed as formula (20).

20 $$yaw = \arctan (\frac{2(wz + xy)}{{1 - 2(y^{2} + z^{2} )}})$$

Then the angle α between ${\text{yaw}}_{\text{A}}$ and ${\text{yaw}}_{\text{B}}$ can be expressed as formula (21):

21 $$\alpha = \arctan (\frac{{2(w_{B} z_{B} + x_{B} y_{B} )}}{{1 - 2(y_{B}^{2} + z_{B}^{2} )}}) - \arctan (\frac{{2(w_{A} z_{A} + x_{A} y_{A} )}}{{1 - 2(y_{A}^{2} + z_{A}^{2} )}})$$

px and py which is outputted from /odom topic represents the coordinates of point A and point B, where the coordinates of point A are (${\text{px}}_{\text{A}}$,${\text{py}}_{\text{A}}$), and the coordinates of point B are (${\text{px}}_{\text{B}}$,${\text{py}}_{\text{B}}$), then the length of line segment AB is shown in formula (22).

22 $$AB = \sqrt {{\text{(px}}_{B} - {\text{px}}_{A} )^{2} + {\text{(py}}_{B} - {\text{py}}_{A} )^{2} }$$

Since the above angle is radian value, and the time of dt is about 20ms, dt is very short, and the moving distance of the robot in this period is very small, so S1 can be approximated by the green arc S2. Making a vertical line to AB from C which intersects AB at point D, and it can be seen that D is the midpoint of AB through geometric relations, then AD=BD=1/2×AB.

Since the above angle is radian value, and the time of dt is 20ms, dt is very short, and the moving distance of the robot in this period is very small, so S1 can be approximated by the green arc S2. Making a vertical line to AB from C which intersects AB at point D, and it can be seen that D is the midpoint of AB through geometric relations, then AD=BD=1/2×AB.

As shown in Fig. 15, it can be seen from the geometric relationship that $\angle {\text{ACB}}$=α, and $\angle {\text{ACD}}$=1/2α, formula (23) can be obtained. So S2 in Fig. 15 can be obtained:

23 $$AC = BC = {\raise0.7ex\hbox{${AD}$} \!\mathord{\left/ {\vphantom {{AD} {\sin \frac{\alpha }{2}}}}\right.\kern-0pt} \!\lower0.7ex\hbox{${\sin \frac{\alpha }{2}}$}}$$

Synthesize formula (21)-(23), the formula (24) can be obtained

24 $$AC = BC = \frac{{\frac{1}{2}\sqrt {{\text{(px}}_{B} - {\text{px}}_{A} )^{2} + {\text{(py}}_{B} - {\text{py}}_{A} )^{2} } }}{{{\text{sin(}}\frac{{1}}{{2}}{(}\arctan (\frac{{2(w_{B} z_{B} + x_{B} y_{B} )}}{{1 - 2(y_{B}^{2} + z_{B}^{2} )}}) - \arctan (\frac{{2(w_{A} z_{A} + x_{A} y_{A} )}}{{1 - 2(y_{A}^{2} + z_{A}^{2} )}}){))}}}$$

Which can be obtained:

25 $$S_{2} = AC\times\alpha = \frac{{1/2\sqrt {(px_{B} - px_{A} )^{2} + (py_{B} - py_{A} )^{2} } }}{{\sin \left( {1/2\left( {\arctan \left( {2(w_{B} z_{B} + x_{B} y_{B} )/1 - 2(y_{B}^{2} + z_{B}^{2} )} \right) - \arctan \left( {2(w_{A} z_{A} + x_{A} y_{A} )/1 - 2(y_{A}^{2} + z_{A}^{2} )} \right)} \right)} \right)}} \times \alpha$$

For dvi, that lx,ly,lz,ax,ay,az in this row can be subtracted from lx,ly,lz,ax,ay,az in the previous row to get dvi. Because S2 represents the distance in about 20 ms, the distance S should be the cumulative sum of S2 as formula (26) shows.

26 $$S = \sum\limits_{{{\text{i}} = 0}}^{i = t} {S2}$$

In Table 6, the robot moves on the surface whose coordinate is x–y plane, no components of motion on the z axis, so the key ‘pz’, ‘lz’, ‘ax’, ‘ay’ should be deleted. The key ‘yaw’ can represents the angle of rotation, so the key ox’, ‘oy’, ‘oz’, ‘ow’ can be deleted. The key ‘power’ represents the power consumption, so the key ‘current’, ‘voltage’ can be deleted. After the above processing, the Table 7 is obtained, in which the key ‘yaw’ is the yaw in formula (20), the key ‘yawdiff’ is the ${\upalpha }$ in formula (21), the key ‘Schange_c’ is the S2 in formula (25) means the distance that robot moves along the curve path in time interval 20 ms, ‘Schange_s’ is the one along straight path in 20 ms, the key ‘distance_c’ is the S in formula (26) which means the distance that robot moves along the curve path in 1 s, ‘Schange_s’ is the one along straight path in 1 s. If the robot moves straight path but not curve path, such as if the robot moves along line segment AB but not arc S2 in Fig. 15, the yaw value is 0 while px, py are not 0. If the robot rotates in place but not moves, the px, py are 0 while yaw is not 0.

In order to compare the power consumption on different moving path, the speed of the robot should keep stable, otherwise, the power consumption is determined not only by path, but also by speed. Because “yawdiff” means the rotated angle of the robot in 20ms, the value of “yawdiff” is nearly the same in each row, so the variance of “yawdiff” is very small. The variance of “power” is determined by the variance of current, different path will lead different current, so the variance of current is large in the moving process, and the variance of “power” is large.

The data in Table 8 comes form the experiment of robot that moves randomly along various path, such as moving along curve line, straight line, or rotating in place.

**Table 8:** The table of values in one second.

Second	px	py	lx	ly	az
1,691,289,815	− 0.064948742	− 0.409979159	0	0	0
1,691,289,816	− 0.064948742	− 0.409979159	0	0	0
1,691,289,817	− 0.064949722	− 0.409982176	0.00016	0	8e− 05
1,691,289,818	− 0.115638127	− 0.564948065	0.28578	8e− 05	− 0.00088
1,691,289,819	− 0.158213113	− 0.68890615	0.021879999999999997	0.0008	0.010959999999999994
1,691,289,820	− 0.143509438	− 0.627549311	− 0.16102	− 0.00088	0.12041999999999999
1,691,289,821	− 0.114134145	− 0.455068063	− 0.19116	− 0.00064	0.11963999999999998
1,691,289,822	− 0.106861958	− 0.216829638	− 0.27274	0.00024	0.12289999999999997
1,691,289,823	− 0.126755151	0.02187789802805419	− 0.14368	0.00016	0.06581999999999998
1,691,289,824	− 0.131185039	0.052635177314834004	0.07314	0.0010400000000000001	− 0.05536
1,691,289,825	− 0.101356816	− 0.234587799	0.29552	− 0.00056	− 0.12578
1,691,289,826	− 0.105215073	− 0.470436822	0.45549999999999996	0.0030399999999999997	− 0.08218
1,691,289,827	− 0.166707348	− 1.120029436	0.708	0.0036	− 0.0805
1,691,289,828	− 0.297630241	− 1.81669801	0.7101000000000001	0.00432	− 0.07926
1,691,289,829	− 0.497284653	− 2.49606227	0.70864	0.0036	− 0.07548
1,691,289,830	− 0.743722121	− 3.113118045	0.495	0.00256	− 0.2709

Second	yaw	yawdiff	SChange	Distance	Power
1,691,289,815	− 1.884955592	0	0	0	3.798288011720836
1,691,289,816	− 1.884955592	0	0	0	2.373929871267378
1,691,289,817	− 1.884955592	0	0	0	2.373929871267378
1,691,289,818	− 1.890889712	0	0.000296962782929056	0.014848139146452801	2.4416000729998406
1,691,289,819	− 1.88425746	0.0003490658504150934	1.8478303883804203e− 05	0.0009239151941902102	7.465175832376489
1,691,289,820	− 1.811302698	0.002443460952784644	0.00046339920617226223	0.02316996030861311	3.4032421588110653
1,691,289,821	− 1.679704872	0.0027925268031699837	0.0006265297339371176	0.03132648669685588	5.011979990105504
1,691,289,822	− 1.542871059	0.002443460952792904	0.0007453624607748275	0.03726812303874137	4.854399944170558
1,691,289,823	− 1.419650814	0.0017453292519873243	0.000452168013358242	0.022608400667912098	6.143752126319526
1,691,289,824	− 1.404291916	− 0.001047198	0.0003313277472621384	0.01656638736310692	1.9048480491034303
1,691,289,825	− 1.527512161	− 0.002443461	0.0011035311170605642	0.055176555853028206	2.265879008096526
1,691,289,826	− 1.621759941	− 0.001745329	0.0008160007576463838	0.040800037882319194	0.35249498211365277
1,691,289,827	− 1.708328272	− 0.002094395	0.0016577427915023702	0.08288713957511852	27.427400594453196
1,691,289,828	− 1.814095225	− 0.002094395	0.0016936253656853115	0.08468126828426556	10.643112933100872
1,691,289,829	− 1.906946741	− 0.001745329	0.0014323015637047523	0.07161507818523762	10.666609970751484
1,691,289,830	− 2.072403954	− 0.005934119	0.0029145922019338743	0.14572961009669375	10.58811953395481

After the data processing of the values in Table 8, only keeping the values of three which are key “distance_c”, “distance_s” and “power”, the values of other keys in Table 8 are deleted from the database, the key “distance_c” can be renamed as “distance along curve path”, key “distance_s” can be renamed as “distance along straight path”.

The data in Table 9 shows the statistical values of data of Table 8, the mean velocity along x axis is much larger than the mean velocity along y axis, it means that the robot rotates more frequently on curve path along x axis than along y axis, and the mean value of yawdiff and Schange is very small, because the changes of yaw value and changes of distance is very small in 20 ms.

**Table 9:** The statistical values of data in Table 8.

	px	py	lx	ly	az
Mean	− 1.535987694	− 1.047626707	− 0.008057834	0.00019606136251026468	− 0.016508854
Variance	3.623633005405085	1.5150362728162912	0.12410324829552889	4.421391021983007e− 06	0.08573606333536214
Std_deviation	1.903584252247608	1.2308680972453105	0.3522829094570568	0.0021027103989810407	0.29280721189096787
Confidence_interval_0.025	− 1.629012513	− 1.107777069	− 0.025273281	9.330559849334969e− 05	− 0.030817828
Confidence_interval_0.975	− 1.442962875	− 0.987476345	0.009157613250088067	0.0002988171265271797	− 0.00219988
Margin_of_error/mean	− 0.060563518	− 0.057415835	− 2.136485747	0.5241000200206977	− 0.866745454

	yawdiff	SChange	Distance	Power
Mean	3.963179514908269e− 05	0.002567487617502416	0.12837445114463578	6.411329527275272
Variance	0.0006298198981196032	9.702748972553719e− 05	0.24256870294752575	24.27496247068035
Std_deviation	0.025096212824241095	0.00985025328230382	0.4925126424240557	4.926962803866125
Confidence_interval_0.025	− 0.001186776	0.0020861230404908863	0.10430622335406697	6.170557508722682
Confidence_interval_0.975	0.0012660396371113676	0.003048852194513946	0.15244267893520458	6.652101545827861
Margin_of_error/mean	30.945048977693634	0.1874846732385757	0.1874845623561952	0.03755414809491379

The variance of px is much larger than variance of py in Table 9, it means the robots changes the positions with larger distance along x axis than along y axis, the variance of power consumption is much larger, because in Fig. 14, the robots stops during a period and starts moving again, the power consumption changes a lots when it starts moving, and the purple power consumption line changes dramatically sometimes in Fig. 14, so the variance of power consumption becomes larger.

The yawdiff is determined by the α and AC in Fig. 15, and α and AC differs a lot in 20 ms, the margin_of_error/mean of yawdiff is very large in Table 9 because the distance is the accumulation of Schange, the distribution of distance and Schange is nearly the same, the margin_of_error/mean of distance and Schange is nearly the same in Table 9.

Table 10 shows the relationship of the values of three kept keys is analyzed with Pearson correlation coefficient, the Pearson correlation coefficient value of power consumption which relates with straight path is lower than that of curve path in Table 10, so there is lower power consumption if robot moves along straight path, that moving along straight path and rotate in place will have less power consumption than moving along curve path, the same result can be obtained from the theoretical analysis in the following section.

**Table 10:** Pearson correlation coefficient of values of three kept keys.

	Distance along straight path	Distance along curve path	Power consumption
Distance along straight path	1.00000	0.37312	0.26899
Distance along curve path	0.37312	1.00000	0.45637
Power consumption	0.26899	0.45637	1.00000

**Fig. 16:**
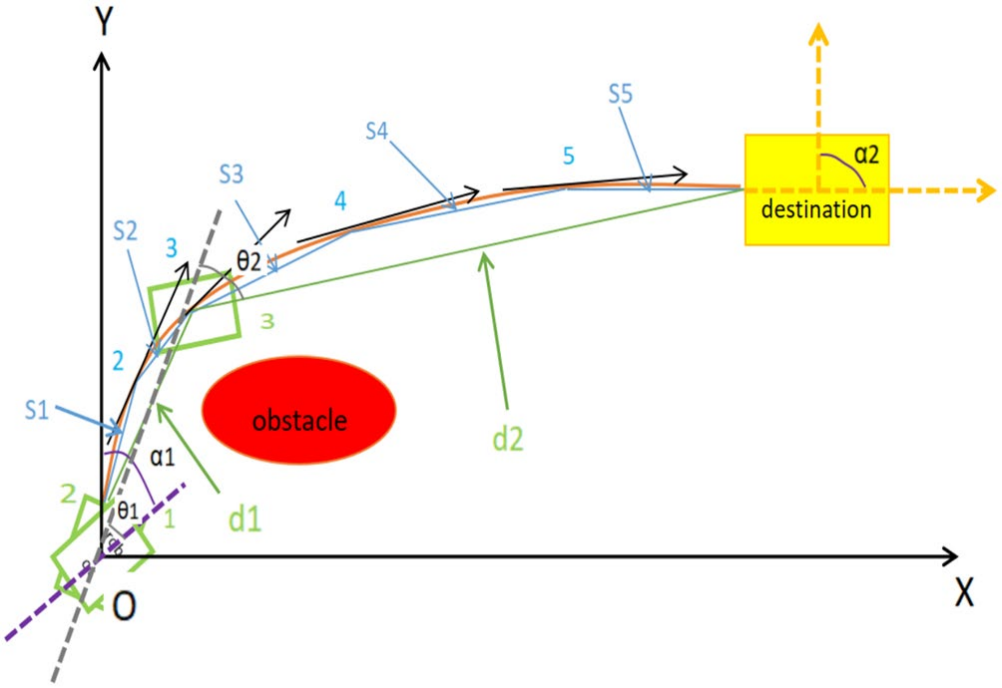
Comparison of power consumption of various paths when the robot avoids obstacles in the dynamic environment.

As shown in Fig. 16, the green rectangle is the mobile robot, the red oval is the obstacle that suddenly appears in front of the robot in the dynamic environment, and the destination is the yellow rectangle. In order to avoid collision with red obstacles, three optional moving paths can be selected: the first is an orange arc, the second is a blue line segment, and the third is a green line segment. The blue line segment can be divided into five segments from S1 to S5, and the green line segment can be divided into two segments d1 and d2.

If the blue line is divided into infinite stages, the length of the orange curve L can be taken as the sum of infinite blue line segments, and the angle of rotation of the robot is the black arrow line which points to the tangential direction of the orange arc. From Fig. 16, the following result can be obtained that L > S1 + S2 + S3 + S4 + S5 > d1 + d2.

Following is the calculation of the total angle of rotation when robot moves along orange arc, green line and blue line respectively, the robot points to purple dotted line at the initiation position, if moving along with orange arc, the robot should rotate α1 to Y axis on left, then moving along the black arrow on the right, when it reaches to the destination, the total angle of rotation γ = α1 + α2. If moving along with green line, robot should rotate θ1 from purple dotted line to gray dotted line first, then rotate θ2 to the destination, the total angle of rotation when along green line is β = θ1 + θ2, because α1 > θ1 and α2 > θ2, then α1 + α2 > θ1 + θ2, so γ > β, and the angle of rotation will be larger if the robot moves along orange curve than along green line. For the blue line, it is obvious that the total angle of rotation along blue line is ɡ, and ɡ > β, and more times rotations will lead to more distance, so the angle of rotation will be less when robot moves along green line.

Compared with the above analysis, when the robot avoids the obstacles in the dynamic environment, that moving along green line, but not blue line or orange curve line, will have less power consumption.

From the experimental and theoretical results, it is obvious that moving straight path and rotating in place will save more power than moving on curve path.

## Navigation with least power consumption

Navigation is the basis of other functions for home service robot, because the robot moves continuously and consumes lots of battery power, how to design the navigation process with least power is important for home service robot.

### Obstacle avoidance of home service robot in the dynamic environment with the least power consumption

According to the above analysis of path planning algorithms, in order to move with least power consumption, the robot should plan the straight path and rotate in place, but not the curve path. When there is an obstacle blocks on the designed path in the dynamic environment, in order to avoid the obstacle with least power consumption, the robot should also moves along straight path and rotate in place, but not moving along curve path to avoid the obstacle.

**Fig. 17:**
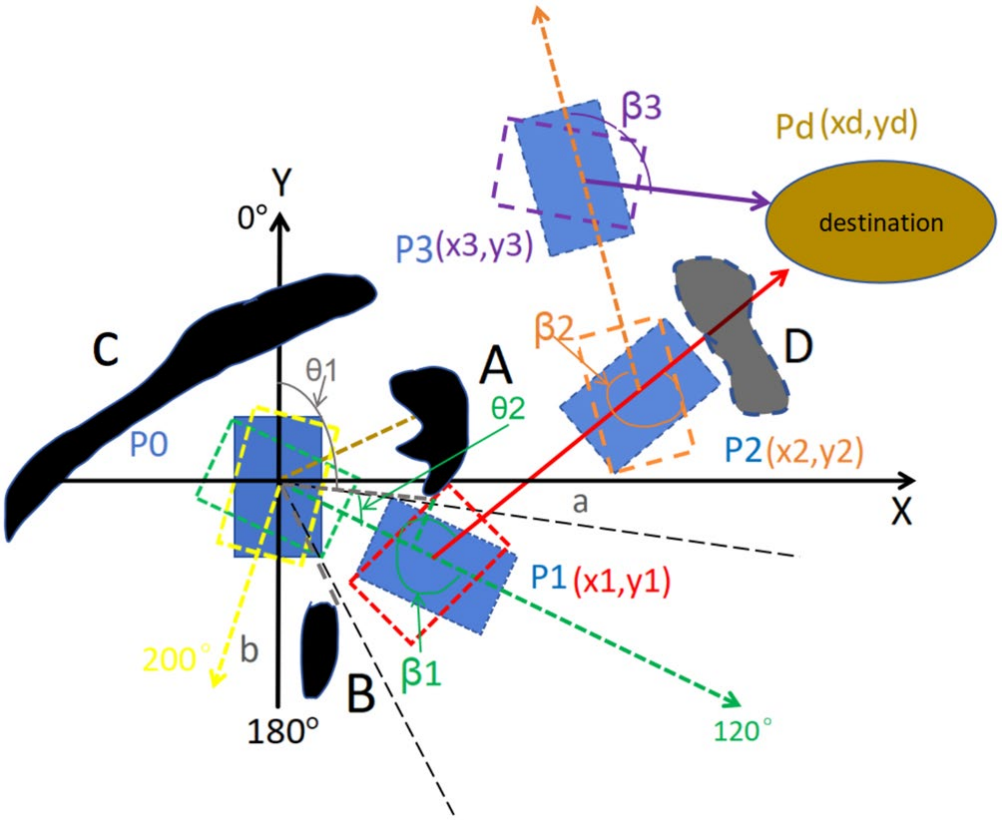
Navigation of a home service robot in an indoor dynamic environment.

In Fig. 17, the robot is a blue rectangle, its initial position is P0, and its initial orientation points to the positive direction of Y axis. The laser scan sensor on the robot scans the surrounding obstacles every second, from the outputted data of the laser scan sensor, it can be seen that the positive Y-axis is 0 degrees, and the negative Y-axis is 180 degrees, there are three obstacles around the robot, namely A, B, C. The destination is a brown circle. The angle γ is the angle which points to the direction that from robot towards to the destination , if the coordinates of P0 is (× 0,y0), on the initial position P0 is (0,0), the coordinates of the destination is (xd,yd), so the angle γ at P0 is the angle between the Y-axis and the brown dotted line, which can be calculated by formula (27).

27 $$\gamma = \arctan \frac{{y_{d} - y_{0} }}{{x_{d} - x_{0} }} = \arctan \frac{{y_{d} }}{{x_{d} }}$$

In Fig. 17, in order to move to the destination with the least power consumption, the robot should rotate angle γ first at P0, then move straightly along the brown dotted line to the destination, but not move curve lines. Now there is an obstacle A appears suddenly on the brown dotted path in the dynamic environment, in order to avoid the obstacle A with least power consumption, the robot needs to rotate in place at P0 and move straightly from the gaps between the obstacles, but not move curve path. Because the laser scan sensor scans the angle and distance of the obstacles in the surrounding environment, it is therefore possible to calculate the distance between obstacles. Usually obstacle can be detected if it is in the scope of 6 m’ scanning range, as shown by the gray dashed line in Fig. 17, if the distance of obstacle is more than 6 m, the laser scan sensor will not be able to detect the obstacle, as shown by the black dashed line in Fig. 17.

### The chosen of the gap between obstacles

**Fig. 18:**
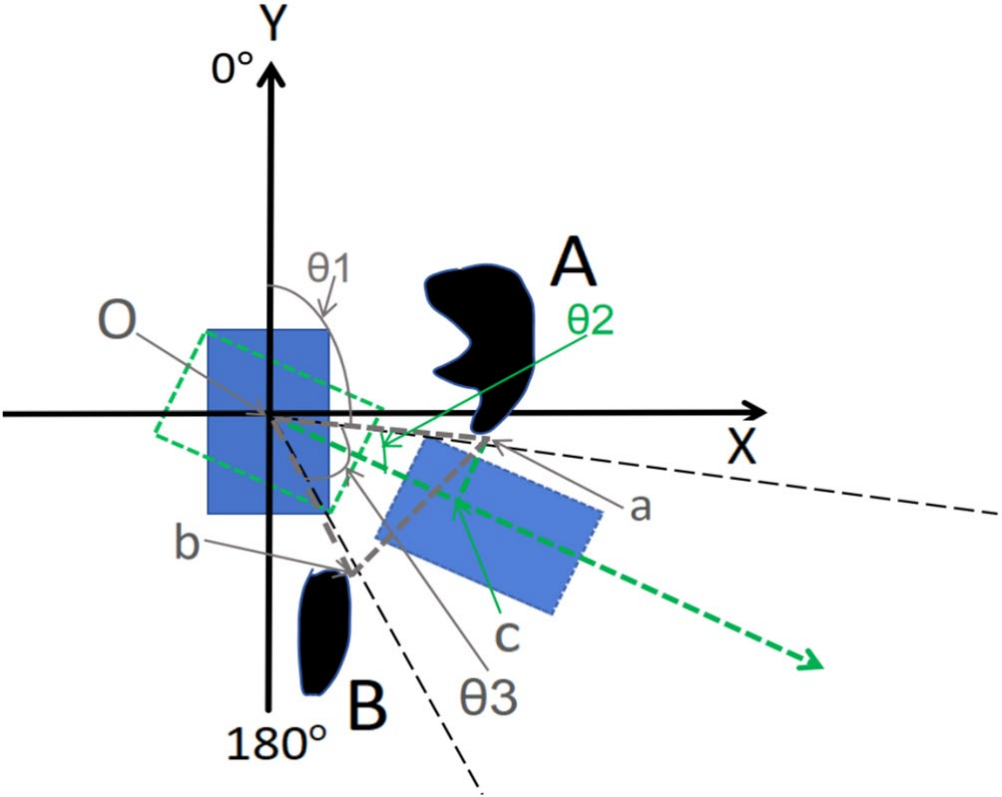
Obstacle avoidance of home service robot in indoor dynamic environment.

In order to avoid the obstacles, the robot should move out from the gaps of obstacles, taking a part of Fig. 17 as Fig. 18 shows, O is the origin of coordinate, the bottom edge of obstacle A is point a, and the top edge of obstacle B is point b.

In Fig. 18, Because the length of the line segments Oa and Ob can be obtained from the laser scan data, and the angle between Oa and Ob can also be obtained from the laser scan data, such as θ3, the length of the line segment ab can be obtained from the cosine formula, as shown in formula (28).

28 $${\text{ab}} = \sqrt {oa^{2} + ob^{2} - 2 \times oa \times ob \times \cos (\theta 3)}$$

Because the width w of the robot can be measured, taking into account redundancy, if w > 1.2 × ab, it means that the robot can pass through the gap between the obstacles, otherwise it can not pass. The following is to judge which gap to pass through and how to pass it.

In Fig. 17, the gap between obstacle AC is smaller than the width of the robot, the gap between obstacle AB and obstacle BC is larger than the width of the robot,so the robot needs to go through the gap between obstacle AB or obstacle BC, compared with gap between the obstacle BC, moving along the direction of the gap between AB is more closer to the destination, which is shown with the green dotted arrow in Fig. 17, the above judgement is to compute the angle between the direction of gap and the brown dotted line, the gap with smaller angle is the correct gap. Therefore, in Fig. 17, in order to avoid the obstacle with least power, the robot should rotate 120°to the right in place first, and then moving out from the gap between AB straightly, that is moving along green dotted arrow line, but not moving curve line to avoid the obstacle A. The following will explain how to obtain 120°which points to the green arrow in Fig. 17.

### The location of angle when robot rotates in place to avoid the obstacle

In Fig. 18, Making a vertical line of the green dotted line from point a, and intersecting with the green dotted line at point c. Considering redundancy, the length of ac should be greater than 0.6 times half of the robot width w, then the angle θ2 between Oa and the green dotted line can be obtained as the formula (29) shows.

29 $$\theta 2 = \arcsin (\frac{{{\text{ac}}}}{Oa}) = \arcsin (\frac{3 \times w}{{5 \times Oa}})$$

The length of the gray dashed line segment Oa and the angle θ1 can be outputted from the laser scan sensor. In Fig. 18, the computed localization of angle of the robot is β0, then β0 = θ1 + θ2, because θ1 can be directly obtained, θ2 can be obtained from formula (29), so β0 can be obtained, in Fig. 18, β0 is 120°, then the robot will start to rotate 120°, the actual localization of angle of the robot can be predicted by the above machine learning method, the robot will check the actual localization of angle of the robot continuously, and the robot will stop rotation when the actual localization of angle reaches 120°, as shown by the rectangle of the green dashed line in Fig. 17.

### Moving straightly from the gap between the obstacles

After the rotation to avoid the obstacle that is blocked on the path whose direction is from robot towards to the destination, the robot starts moving along the green arrow line straightly, if the robot stops at point P1, it is necessary to obtain the moving distance from P0 to P1 in Fig. 17.

In Fig. 18, the point P is a point on the green arrow line whose coordinate is (x,y), and the length OP represents the moving distance, so coordinate (x,y) can be expressed as (OP × sin(θ1 + θ2),OP × cos(θ1 + θ2)). In Fig. 17, the destination direction angle γ at point P can be expressed as formula (30), whose direction points to the red arrow line.

30 $$\gamma = \arctan \frac{{y_{d} - y_{{}} }}{{x_{d} - x_{{}} }}$$

It is necessary to judge whether there are obstacles in the direction which points to angle γ continuously, that is checking if there is continuous distance’s data points which more than 1 m within the angle’s range scope of γ ± 40° which can be judged from the laser scanning data, if the check result is true, the robot should continue to move straightly along the green dotted line, otherwise, the robot should stop and needs to rotate again. In Fig. 17, the robot should stop at point P1 where no obstacles within γ ± 40°, the angle of rotation is β1, which can be expressed by the formula (31).

31 $$\beta_{1} = \gamma { - }\beta_{0} + 2\pi = \arctan \frac{{y_{{\text{d}}} - y_{1} }}{{x_{d} - x_{1} }} - \theta_{1} { - }\arcsin (\frac{3 \times w}{{5 \times Oa}}) + 2\pi$$

If the calculated β1 is less than 0, the result should be added 2 $\uppi$, if β1 is greater than 180 degrees, the robot should rotates 360°-β1 on left, otherwise it rotates β1 on right. After the robot rotates β1, the localization of angle of the robot points to the direction of the red arrow in Fig. 17, and then the robot continues moving along the direction of the red arrow. Because obstacles can move in the dynamic environment, when the robot moves to the position of P2, suddenly an obstacle D appears in front of the robot and blocks on the path to the destination, according to the above analysis, the robot should rotate 360°-β2 to the left, and then moving straightly to the position of P3 now. At this time, it can be seen that the value of γ is between 90° and 270°, which means that the destination has been behind the robot. If no obstacle is detected within the range of 1 m in the angle scope of γ ± 40°, the robot should continue to move straightly along the orange dotted line, which is the direction of β2, otherwise, the robot needs to rotate, the angle of rotation is β3, which can be expressed by the formula (32), and the above process is iterated until the destination is finally reached.

In the above process, the total moving distance of the robot can be expressed by formula (31).

32 $$S = \sum\limits_{i = 1}^{n} {\sqrt {({\text{x}}_{{\text{i}}} - x_{i - 1} )^{2} + ({\text{y}}_{{\text{i}}} - y_{{\text{i - 1}}} )^{2} } }$$

The above method realizes the navigation of the home service robot with the least power consumption in the indoor dynamic environment, the robot finishes the navigation process only by moving straightly and rotate in place, not by moving curve paths, which can save more power consumption.

## Experiment

There are some existed navigation algorithms, DWA ( Dynamic Window Approach ) and TEB ( Time-Elastic Band Algorithm ) are two distinct local path planning algorithms, suitable for dynamic obstacle avoidance and path optimization in robot navigation, each with its own characteristics.

DWA algorithm is proposed based on speed space sampling, by generating multiple sets of speed combinations (v, ω) and simulating path prediction, the optimal path is finally selected. It is suitable for differential drive robots (such as two-wheel robots), but it is prone to getting stuck in local optima (such as U-shaped obstacle scenarios).

TEB algorithm is proposed by adjusting the time elastic band to optimize the path, it takes into account both path smoothness and obstacle avoidance efficiency. It is suitable for robots with Ackerman steering structures (such as car models), which can optimize time efficiency and posture adjustment, but the computational load is large, and it may cause reverse adjustment of the posture.

In order to compare the power consumption of the existed navigation algorithm and the proposed navigation algorithm in this paper, the experiment is conducted that the navigation process is completed with DWA, TEB and the proposed navigation algorithm separately in the home where is a dynamic indoor environment.The DWA and TEB algorithm can be selected in the launch file in ROS system, which can be loaded before the navigation process started.

**Fig. 19:**
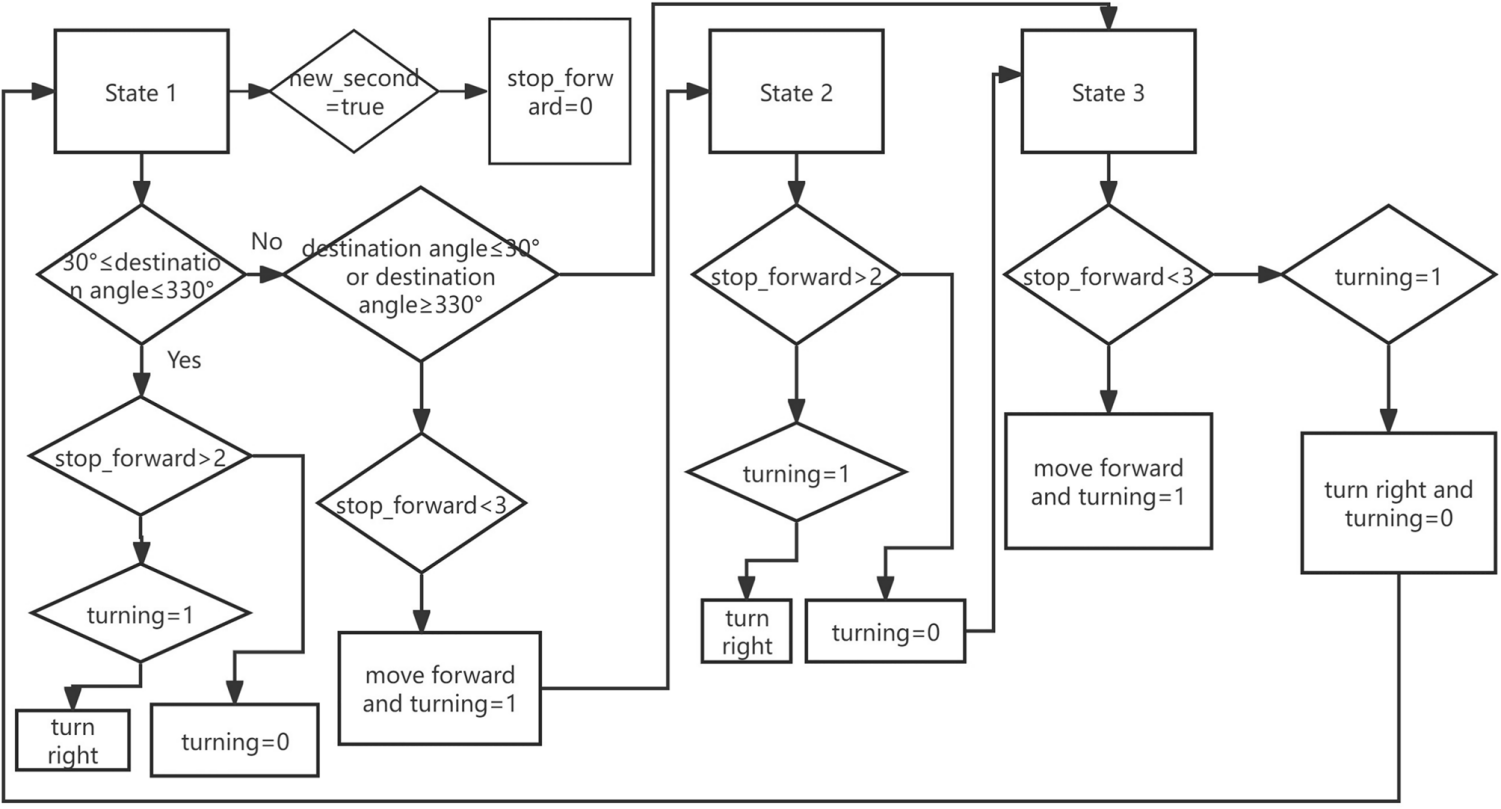
The logic chart of the programming of the proposed navigation algorithm.

For the proposed navigation algorithm in this paper, the logic chart of the programming can be listed as Fig. 19 shows.

According to the introduction of the above Sect. 4 in this paper, the programming logic model can be designed as a state machine model that switches between three states, the state machine runs until the mobile robot arrives the destination. The destination angle in Fig. 19 is the angle between orientation of robot and the destination, which is the β in Fig. 17. When the laser scanning scope is within the range of 330°to 30° and there is a moving obstacle within 1 m from the laser scanning sensor, the variable “stop_forward” will increase by 1, when a new second arrives, the variable “stop_forward” will be 0. When variable “turning” is 1, the robot can turn right, otherwise, it can not turn right.

**Fig. 20:**
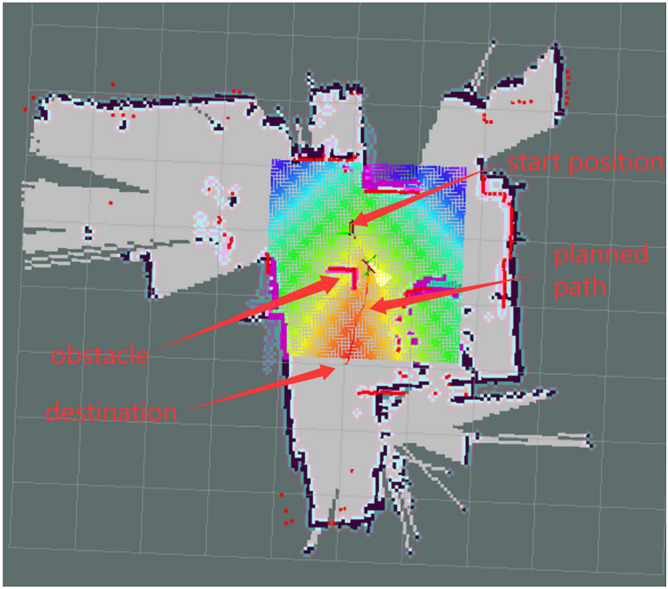
The navigation process in ROS.

The navigation is tested with DWA, TEB and proposed navigation algorithm separately in the home, there is no obstacle when building the map, after the map is built, an obstacle is placed in front of the robot in the test, the start position of the robot, the destination of the robot and the position of obstacle are totally the same in the test of DWA, TEB and the proposed navigation algorithm, the navigation process is shown in ROS as Fig. 20 shows. The power consumption and all other values can be obtained according to the introduction in Section "Path planning with the least power consumption.", when the values of lx, ly and az are all 0, it means the robot stops in the navigation process, and this row should be deleted in database, the left values are the valid data which are shown as Tables 11, 12, 13 shows.

**Table 11:** All the data in one second with DWA algorithm.

Second	px	py	oz	lx	ly	az	Voltage	Current	Power
1,751,513,539	− 0.024989074	0.28825564658620695	0.018352882982619997	− 0.120965517	− 0.002206897	0.17920689655172414	10.59300041	0.379000008	4.014747242014861
1,751,513,540	− 0.166463801	− 0.029244092	0.1719523545703	− 0.19622	0.00256	0.49085999999999996	10.60700035	0.375	3.977625132
1,751,513,541	− 0.329908648	− 0.132337458	0.35388211214000004	− 0.19596	0.0015199999999999999	0.12976000000000001	10.60200024	0.75	7.951500177
1,751,513,542	− 0.459453148	− 0.247985106	0.348724052	− 0.14488	0.0004	− 0.04664	10.48799992	0.649999976	6.817199695410106
1,751,513,543	− 0.565207588	− 0.338976002	0.35461035459999996	− 0.1396	0.00024	0.03392	10.44099998	0.537	5.606816996764257
1,751,513,544	− 0.666648388	− 0.432645653	0.381935086	− 0.13652	− 0.00024	0.15398	10.46899986	0.561999977	5.883577678229218

**Table 12:** All the data in one second with TEB algorithm.

Second	px	py	oz	lx	ly	az	Voltage	Current	Power
1,751,513,539	− 0.024989074	0.28825564658620695	0.018352882982619997	− 0.120965517	− 0.002206897	0.17920689655172414	10.59300041	0.379000008	4.014747242014861
1,751,513,540	− 0.166463801	− 0.029244092	0.1719523545703	− 0.19622	0.00256	0.49085999999999996	10.60700035	0.375	3.977625132
1,751,513,541	− 0.329908648	− 0.132337458	0.35388211214000004	− 0.19596	0.0015199999999999999	0.12976000000000001	10.60200024	0.75	7.951500177
1,751,513,542	− 0.459453148	− 0.247985106	0.348724052	− 0.14488	0.0004	− 0.04664	10.48799992	0.649999976	6.817199695410106
1,751,513,543	− 0.565207588	− 0.338976002	0.35461035459999996	− 0.1396	0.00024	0.03392	10.44099998	0.537	5.606816996764257
1,751,513,544	− 0.666648388	− 0.432645653	0.381935086	− 0.13652	− 0.00024	0.15398	10.46899986	0.561999977	5.883577678229218

**Table 13:** All the data in one second with the proposed navigation algorithm.

Second	px	py	oz	lx	ly	az	Voltage	Current	Power
1,751,513,539	− 0.024989074	0.28825564658620695	0.018352882982619997	− 0.120965517	− 0.002206897	0.17920689655172414	10.59300041	0.379000008	4.014747242014861
1,751,513,540	− 0.166463801	− 0.029244092	0.1719523545703	− 0.19622	0.00256	0.49085999999999996	10.60700035	0.375	3.977625132
1,751,513,541	− 0.329908648	− 0.132337458	0.35388211214000004	− 0.19596	0.0015199999999999999	0.12976000000000001	10.60200024	0.75	7.951500177
1,751,513,542	− 0.459453148	− 0.247985106	0.348724052	− 0.14488	0.0004	− 0.04664	10.48799992	0.649999976	6.817199695410106
1,751,513,543	− 0.565207588	− 0.338976002	0.35461035459999996	− 0.1396	0.00024	0.03392	10.44099998	0.537	5.606816996764257
1,751,513,544	− 0.666648388	− 0.432645653	0.381935086	− 0.13652	− 0.00024	0.15398	10.46899986	0.561999977	5.883577678229218

From the values of Table 14, the following can be obtained, the mean of power consumption of proposed navigation algorithm is lower than that of DWA and TEB, it means that the proposed navigation algorithm can complete the navigation with least power, the reason has been analyzed in Sect. 4 in this paper. DWA algorithm can predict many potential paths and this needs much more calculation of CPU and will consume more power consumption than TEB and proposed navigation algorithm.

**Table 14:** The statistical values of power consumption in Tables 11, 12, 13.

	DWA	TEB	Proposed navigation algorithm
Mean of power consumption	6.609075177601161	6.549646949861281	6.448674024026799
Variance of power consumption	2.4372783813998486	0.7827173436071639	1.088270709735845
Std_deviation of power consumption	1.5611785232316797	0.8847131419885002	1.0432021423175113
Confidence_interval_0.025 of power consumption	5.835991947110742	6.136985238751943	6.115308266251999
Confidence_interval_0.975 of power consumption	7.3821584080915805	6.96230866097062	6.782039781801599
Margin_of_error/mean	0.1169729818039411	0.06300518398447084	0.051695240995703697

The variance and std_variance value of power consumption of TEB is lower than that of DWA and proposed navigation algorithm,the planned path of TEB algorithm is a smooth curve path, and the robot moves smoothly continuously in each second, so the variance and std_variance value is smaller. For the DWA algorithm, it calculates many potential paths and select one path according to the configured priority in the launch file first, which consumes lots of power consumption in this stage, then the robot move along the selected path and no calculation of paths at this moment, the above process continues and the changes of power consumption becomes dramatically, so the variance and std_variance value of power consumption of DWA is higher than others.

From the confidence interval and margin of error, it shows that more values are integrated around the mean value for the proposed navigation algorithms than that of DWA and TEB algorithms. Because there are only two actions in the proposed navigation algorithm, that is move straight path and rotate in place, the power consumption of move straight path and rotate in place is different, so the variance and std_variance value of power consumption of proposed navigation algorithm is higher than TEB algorithm. For TEB algorithm, sometimes the robot needs to move a curve path with large angle and small radius, which needs the large driving force and consumes lots of power consumption at this moment, and this power consumption will surpass the mean value at this moment, compare with the only two actions of proposed navigation algorithm, the confidence interval and margin of error of TEB is higher. For DWA, the power consumption of calculation of many paths needs to be considered, the confidence interval and margin of error of DWA will be more higher than TEB and proposed navigation algorithm.

From the above analysis of the result of experiment, the mean power consumption of proposed navigation algorithm is the least, for the variance and std_variance value, confidence interval and margin of error value of power consumption, the proposed navigation algorithm performs good, and this will save the power consumption of battery and extend the battery life.

## Conclusion

In this paper, five novel points are proposed, the first is the localization of position of home service robot with least power consumption, the geometrical relationship of the robot and home appliances is analyzed to calculate the location of position of the robot, avoiding the complex computation of large feature data points of SLAM algorithm continuously, which saving the workload of CPU and RAM, thus saving the power consumption of the battery of the robot.

The second novel point is that the localization of angle of home service robot in the indoor environment, after the analysis from three machine learning algorithms, as well as the error from the photoelectric encoder and friction on the ground, providing a model to obtain the localization of angle, which also save power consumption of CPU and RAM that come from the complex SLAM algorithms frequently.

The third novel point is that two supplements and optimizations are proposed for the previous data processing algorithms, the first is to obtain the power consumption when robot is stopped, and the power consumption that only for the movement of the robot is computed in the database, the second is that the noise data should be judged and removed before selecting the abnormal data in the database.

The fourth novel point is that a conclusion is obtained for the path planning with least power consumption, which can be concluded not only from the data processing in the database, but also from theoretical analysis, both of them can concludes that moving on straight path and rotating in place will save more power than moving on curve path.

The fifth novel point is that the navigation algorithm with least power consumption for the home service robot in the indoor dynamic environment, in which the obstacle avoidance in the dynamic environment, the computation of angle between robot and destination, the iterated navigation process, all of them are analyzed based on the result from the above localization and path planning algorithm.

The conclusions can be applied in the navigation of home service robot, logistics robot and other robot that works in the indoor dynamic environment, to save more power of the batteries, thus to improve the working efficiency and service quality of the robot.

## Supplementary Information

Supplementary Information 1

Supplementary Information 2

Supplementary Information 3

Supplementary Information 4

Supplementary Information 5

Supplementary Information 6

Supplementary Information 7

Supplementary Information 8

## Data Availability

The data that support the findings of this study are available from the Figshare (https://figshare.com) website: The data from /odom topic in ROS(Robot Operation System), the linkage is : https://figshare.com/articles/dataset/Data_from_odom_topic_in_ROS_Robot_Operation_System_/28602410?file=53020430. The data from /battery topic in ROS(Robot Operation System), the linkage is : https://figshare.com/articles/dataset/Data_from_battery_topic_in_ROS_Robot_Operation_System_/28602413?file=53020433. The data of Tables in this manuscript, the linkage is : https://figshare.com/articles/dataset/The_data_of_Tables_in_the_manuscript_/28608212. The above data has also been uploaded in the Supplementary material section in the submission system.
